# Outcome measures in prehabilitation interventions for total hip and knee arthroplasty: A scoping review

**DOI:** 10.1177/02692155251378374

**Published:** 2025-09-19

**Authors:** Nicola Burgess, Stefanie N Voelker, Belinda Phillips, Marnie Graco, Sue Berney, Linda Denehy, Lara Edbrooke

**Affiliations:** 1 Department of Physiotherapy, Melbourne School of Health Sciences, 85084 University of Melbourne, Melbourne, VIC, Australia; 2 Department of Physiotherapy, 3805 Austin Health, Melbourne, VIC, Australia; 3 Department of Anesthesia and Perioperative Medicine, 2538 Monash Health, Melbourne, VIC, Australia; 4 School of Clinical Sciences, 2541 Monash University, Melbourne, VIC, Australia; 5 Institute of Breathing and Sleep, 3805 Austin Health, Melbourne, VIC, Australia; 6 Department of Health Services Research, 3085 Peter MacCallum Cancer Centre, Melbourne, VIC, Australia; 7 Sir Peter MacCallum Department of Oncology, 85084 University of Melbourne, Melbourne, VIC, Australia

**Keywords:** Total knee arthroplasty, total hip arthroplasty, prehabilitation, outcome measures, osteoarthritis

## Abstract

**Objective:**

This scoping review aimed to map outcome measures collected in randomised controlled trials investigating prehabilitation interventions in total hip and knee arthroplasty, and timepoints of collection.

**Data sources:**

A systematic search of MEDLINE, EMBASE, Web of Science, Cochrane, and CINAHL was conducted.

**Methods:**

This review followed the Joanna Briggs Institute Scoping Review methodology. Outcome domains, concepts of interest and assessment tools were characterised using the International Society for Pharmacoeconomics and Outcomes Research Framework, and timepoints for data collection were extracted.

**Results:**

Ninety-two trials (published between June 2001 and March 2025) were included. Most delivered unimodal prehabilitation, with exercise the most common intervention (*n* = 37). The review identified 36 outcome concepts measured with 219 assessment tools. Patient-reported outcomes were collected in 92% of trials (*n* = 85), and was the most heterogenous domain with 102 assessment tools. Performance-based outcomes, most commonly muscle strength, were collected in 66% of trials (*n* = 61) and utilised 47 different tools. Observer-reported outcomes were reported in 60% of trials (*n* = 55), with healthcare utilisation (e.g. hospital length of stay) the most common concept. Clinician-reported outcomes were reported in 48% of trials (*n* = 44) and most frequently included post-operative complications. Biomarker outcomes were rare (*n* = 7, 8%). Timing of outcome collection varied, with just over half the trials collecting both a pre-operative and post-operative timepoint.

**Conclusion:**

This review identified significant variability in outcome measures collected in prehabilitation trials for total hip and knee arthroplasty, highlighting the need for a core set of assessments to facilitate consistent reporting and robust meta-analyses of prehabilitation efficacy.

## Introduction

Total joint arthroplasty is the main therapeutic approach to end-stage hip or knee osteoarthritis. Whilst these surgeries are largely successful, waitlists tend to be long, with patients often waiting over 12 months between initial orthopaedic consultation and surgery.^1^ Whilst waiting for surgery, many patients experience progression of their disease and a significant deterioration in their physical function and quality of life,^2^ which in turn can affect postoperative outcomes.^3,4^

Prehabilitation is the delivery of pre-operative interventions aiming to enhance functional capacity and physiological reserve to withstand surgical stressors and improve postoperative outcomes.^5^ Whilst the value of prehabilitation in improving post-operative outcomes has been well established in several populations including lung cancer and colorectal surgeries,^6,7^ there remains uncertainty around the efficacy of prehabilitation in total hip and knee arthroplasty. While some studies suggest prehabilitation can improve pain, quality of life and functional outcomes and reduce length of stay following total hip or knee arthroplasty, others have demonstrated minimal effect in improving postoperative outcomes in this cohort.^8–10^ This discrepancy is primarily owing to poor methodological quality of studies and significant heterogeneity in outcome measures used to evaluate interventions.^9,11^ There is a need for alignment of outcome collection across trials to allow combined data sets and generate robust evidence.

In 2017, the Outcome Measures in Rheumatology Clinical Trials-Osteoarthritis Research Society International total joint replacement special interest group published a core domain set for outcome types that should be collected in all trials studying total hip and knee arthroplasty.^12^ However, to date there are no recommendations regarding instruments to evaluate each of the domains. Before developing a core set of outcome measures in prehabilitation trials for this cohort, it is vital to have a comprehensive overview of current reporting practices in prehabilitation trials for total hip and knee arthroplasty.

While previous work has been undertaken to examine the reporting of outcome measures used in prehabilitation trials across all surgical populations,^13^ there are no reviews to our knowledge that have mapped the use of outcome measures in prehabilitation trials specifically in the orthopaedic cohort, nor reported how prehabilitation is defined for this population. Therefore, this scoping review aimed to map the outcome measures used in clinical trials of prehabilitation interventions for total hip and knee arthroplasty to understand *what* outcomes are being measured (i.e. outcome domains), *how* they are being measured (i.e. specific tools being used) and *when* they are being measured (i.e. timepoints pre- and post-operative). Secondary aims included analysing the alignment of outcome reporting with the Outcome Measures in Rheumatology Clinical Trials-Osteoarthritis Research Society International core domain set, understanding how prehabilitation is being defined in this population and identifying characteristics of prehabilitation interventions in included trials.

## Methods

The methodology for this scoping review followed the Johanna Briggs Institute scoping review guidelines,^14^ and reporting followed the Preferred Reporting Items for Systematic Reviews and Meta-Analysis extension for Scoping Reviews framework.^15^ The study protocol was prospectively registered at Open Science Framework (https://osf.io/qzxwk). This was updated in November 2024 (prior to data extraction) to reflect changes made to the inclusion and exclusion criteria to ensure only the most relevant studies were included in the review.

### Eligibility criteria

Studies were included if they: (a) were published in full-text format since 2000 and available in English; (b) were randomised controlled trials; (c) included adults undergoing primary elective total hip or knee arthroplasty; (d) had >80% of participants diagnosed with osteoarthritis and (e) investigated the effect of prehabilitation on pre- or post-operative efficacy outcomes for people undergoing total hip/knee arthroplasty. The definition for prehabilitation was: ‘a process from diagnosis to surgery, consisting of one or more pre-operative interventions of exercise, nutrition, psychological strategies and respiratory training, that aims to enhance functional capacity and physiological reserve to allow patients to withstand surgical stressors, improve post-operative outcomes, and facilitate recovery’,^5^ and delivered at least 7 days prior to surgery.^16^ Additionally, trials including education interventions involving multiple elements (i.e. not solely a brochure) were also included, recognising the role education plays in universal prehabilitation.^17^ Studies were excluded if they: (a) included unicompartmental, hemiarthroplasty, bilateral surgery, revision surgery, or >20% of the population were diagnosed with conditions other than osteoarthritis (e.g. rheumatoid arthritis); (b) delivered an intervention pre- and post-operatively without measuring any outcome following the pre-operative portion of the intervention; or (c) the intervention only involved isolated preoperative risk management (e.g. anaemia correction or smoking cessation).

### Identification and selection of studies

A systematic search was conducted on the 9th of May 2024 by one author (NB) in Medline (Ovid), CINAHL (EBSCO), Cochrane CENTRAL (Wiley), EMBASE (Ovid), and Web of Science Core Collection (Science Citation Index Expanded 1900 to present). The search was updated using the identical strategy on 15 May 2025. The search strategy was developed with the assistance of a medical librarian using relevant Medical Subject Headings terms and keywords for main concepts. A Cochrane randomised controlled trial filter^18^ and a date limiter (2000 to present) was used in the search strategy to identify the most relevant trials. See eTable 1 for detailed search strategy and registered protocol for further details (https://osf.io/qzxwk).

Identified citations were uploaded to EndNote version 21 and duplicates removed. Citations were uploaded to Covidence (www.covidence.org/) and screened for eligibility. First, two independent investigators (NB and SV) screened at the title and abstract level. Secondly, full-text examination was completed by the same two authors. Additional investigators (LD, LE, SB and MG) were available to facilitate consensus, if required. Citation searching (forward and backward) was performed on included papers to identify any additional trials.

### Data extraction and analysis

For included citations, data were extracted from the main manuscript, referenced protocols and Supplementary Material by two independent investigators (NB and SV or BP) using a custom data extraction tool in Microsoft Excel. This tool was adapted from a previous scoping review of prehabilitation across all surgical populations.^13^ Study characteristics including sample size, surgical population and intervention components (e.g. program type, duration and frequency) were extracted, as well as participant data used for risk assessment (e.g. comorbidities). For trials combining pre- and post-operative interventions, only the pre-operative portion of the intervention and associated data was analysed in this review.

Outcome data were classified according to the International Society for Pharmacoeconomics and Outcomes Research outcome assessments framework,^19^ which categorises health outcomes into performance-based, clinician-reported, patient-reported, observer-reported, and biomarker outcomes. Definitions of the categories of the International Society for Pharmacoeconomics and Outcomes Research outcome assessments framework and examples are provided in eTable 2. For multidimensional outcomes with components crossing multiple categories, it was first determined if all components were collected (e.g. all trials in the included review that reported the Knee Society Scale collected only the objective knee and function scores and therefore was categorised as a clinician-reported outcome), and secondly who completed the outcome (i.e. clinician, researcher or patient). For each type of outcome, concepts of interest and their specific outcome assessment tools were identified. The International Society for Pharmacoeconomics and Outcomes Research defines the concept of interest as the specific aspect of health or disease that the outcome assessment aims to measure, whereas the outcome assessment itself refers to the instrument used to generate a rating or score that reflects an element of the patient's medical or health status.^19^ For example, health-related quality of life is a concept of interest within patient-reported outcomes, and the 36-item short form survey would be an assessment tool. Where a multi-item outcome tool consisted of items that could be classified under different concepts of interest (e.g. EuroQol-5 Dimension has items that assess the concepts of pain, function and psychological status) and was used in its entirety, the overall purpose of the tool was considered and consensus sought regarding which was the most appropriate concept of interest to classify the tool to avoid double counting (e.g. quality of life).

Timepoints for outcome collection were categorised into pre-operative, admission, ≤30 days post-operative, >30 days to 90 days post-operative and >90 days post-operative. Admission was defined as the time from hospital admission until discharge from hospital care.

Extracted data were also categorised according to the 2017 Outcome Measures in Rheumatology Clinical Trials-Osteoarthritis Research Society International core domain set for total joint replacement.^12^ This includes six core domains (pain, function, satisfaction, revision surgery, adverse events, and death) and three outer domains (patient participation, costs, and range of motion). This categorisation enabled assessment of how well outcome reporting in the included trials aligned with the core domain set. Alignment was defined as the proportion of the nine domains included in the core domain set reported in each study. Mean alignment was calculated separately for studies published before and after the 2017 core domain set release to evaluate changes in outcome reporting. When multi-item instruments (e.g. Oxford Hip Score) were used, authors consulted the referenced instrument to determine what domains were included. If not otherwise specified, it was assumed the full instrument was used and mapped accordingly (e.g. Oxford Hip Score was categorised as both ‘pain’ and ‘function’ in the core domain set). Ambiguities around the ‘satisfaction’ and ‘participation’ domains were addressed through consensus: satisfaction included any outcome (or items within a composite outcome) assessing satisfaction with the intervention or surgery, and participation included any outcome (or items within a composite outcome) related to involvement in life or social activities per the International Classification of Functioning, Disability and Health model^20^ (e.g. sport and recreation domain of the Hip/Knee Osteoarthritis Outcome Scores).

Cost analysis was categorised into intervention costs (e.g. staffing, equipment), hospital-related costs (e.g. admission, inpatient rehabilitation), community costs (e.g. general practitioner visits, outpatient therapy) and personal expenses (e.g. travel, productivity loss).

Cross-checking of all data was performed, and any disagreements resolved through discussion. For quantitative data, descriptive and summary data were analysed in Microsoft Excel.

Qualitative data collection included verbatim definitions of prehabilitation where available. Qualitative data were analysed independently by two coders (NB and SV) using directed content analysis,^21^ which involved reading and coding prehabilitation definition components (as words or small phrases) according to pre-specified categories guided by the Template for Intervention Description and Replication (TIDieR) 12 item checklist for describing interventions.^22^ Similar methodology was previously used to develop the broader prehabilitation definition, applicable to multiple surgery types.^5^

## Results

### Search results

**Figure 1.**
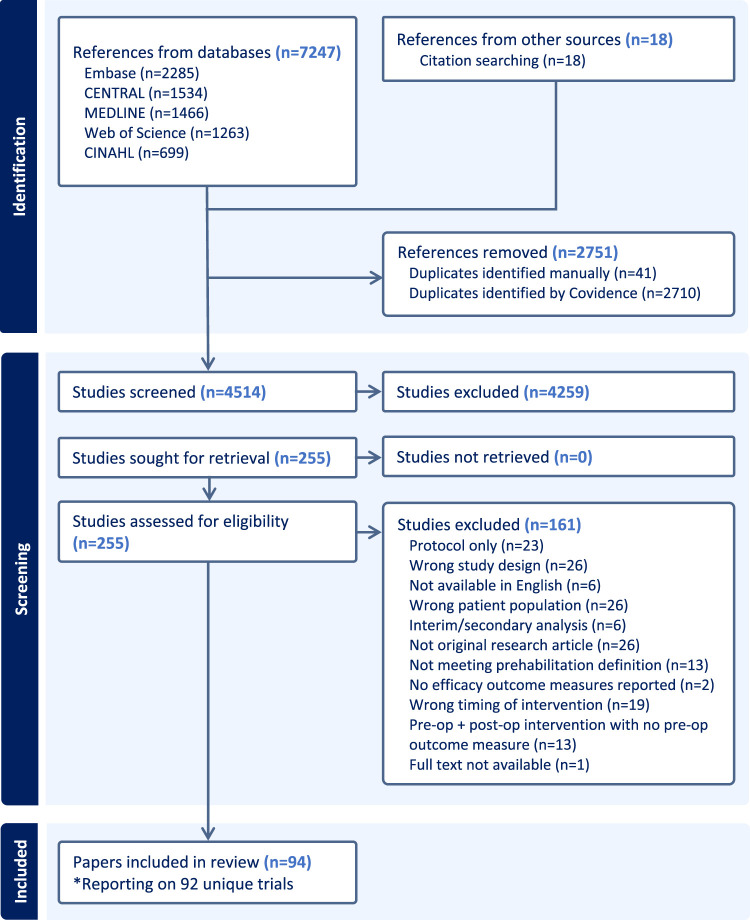
Preferred reporting items for systematic reviews and meta-analyses flow diagram.

The search and screening process is presented in Figure 1. In summary, 4514 records were screened for eligibility. After title and abstract screening, 255 papers underwent full-text review, of which 94 papers (published between June 2001 and March 2025) were included representing 92 unique trials.^23–116^

Pre-op + post-op: intervention delivered both pre- and post-operatively.

*For the purpose of the scoping review, Buvanendran et al.^33^ reported on two separate samples and different interventions within the one paper (Phase 1 and Phase 2) and was therefore treated as two independent trials. Calatayud et al.^34^ and Casana et al.,^36^ Skoffer et al.^96^ and Skoffer et al.,^97^ and Villadsen et al.^110^ and Fernandes et al.^46^ reported on the same sample and intervention and were therefore treated as three trials.

### Trial characteristics

The 92 trials included a total of 6684 participants, with an average sample size of 72.7 (range: 10–336, SD: 55.7). Fifty-five trials (60%) involved participants undergoing total knee arthroplasty, 18 (20%) involved total hip arthroplasty, and 19 (21%) included both surgical types. Across the 92 trials, 5015 participants were eligible but declined to participate, representing 43% of the total eligible population.

An upward trend in number of papers published over time was observed, with almost 40% (*n* = 34/94, 39%) of included papers published in the last five years. Included trials were conducted across 32 countries. Most trials were conducted in Europe (*n* = 40/92, 43%), the United States (*n* = 14/92, 15%), and the United Kingdom (*n* = 13/92, 14%). See eTable 4 for further breakdown.

Individual characteristics and references of included studies are presented in eTable 5.

### Outcome measures according to the International Society for Pharmacoeconomics and Outcomes Research outcome assessments framework

Of the 92 included trials, 68 (74%) reported one primary outcome, four (4%) reported more than one, and 20 (21%) did not specify a primary outcome. Of trials reporting a primary outcome, patient-reported outcomes were the most common followed by performance-based outcomes (Table 1). Nine trials (10%) reported feasibility measures as the primary outcome.

**Table 1.** Types of reported outcome assessments according to the International Society for Pharmacoeconomics and Outcomes Research outcome assessment framework.

ISPOR framework outcome domains	Trials reporting at least one outcome n (%)	Identified as primary outcome measure n (%)	Total times reported across trials^a^ (n)	Number of times reported per trial Mean (SD)	Most common concepts of interest n (%)^b^	Total times concept of interest reported across trials (n)^c^	Most common outcome assessment tools n (%)^d^		Total outcome assessment tools
Patient-reported outcomes	85 (92.4)	39 (41.1)	286	3.4 (1.9)	Disease-specific assessments 57 (67.1)	70	WOMAC	30 (42.9)	102
							KOOS	11 (15.7)	
							Oxford Knee Score	9 (12.9)	
					Pain 44 (51.8)	49	Visual analogue scale	19 (38.8)	
							Numerical rating scale	14 (28.6)	
							KOOS/WOMAC pain only	3 (6.1)	
					Quality of life 37 (43.5)	48	EQ-5D	20 (41.7)	
							SF-36/SF-12	18 (37.5)	
							ICECAP	3 (6.3)	
Performance-based outcomes	61 (66.3)	22 (23.2)	225	3.5 (2.5)	Functional tests 43 (70.5)	65	Timed up and go	22 (33.8)	47
							Stair climbing	18 (27.7)	
							30 s sit-to-stand	13 (20)	
					Muscle strength 30 (47.6)	57	Knee extension	26 (45.6)	
							Knee flexion	11 (19.3)	
							Hip abduction	6 (10.5)	
					Range of motion 24 (38.1)	44	Knee flexion	22 (50)	
							Knee extension	19 (43.2)	
							Hip abduction/flexion/ER	1 (2.3)	
Observer-reported outcomes	55 (59.8)	2 (2.1)	145	1.6 (2.0)	Healthcare utilisation 40 (72.7)	79	Length of stay	37 (46.8)	30
							Decision to delay surgery	14 (17.7)	
							Rehabilitation setting	8 (10.1)	
					Anthropometrics 15 (26.8)	26	BMI	9 (34.6)	
							Weight	8 (30.8)	
							Height	3 (11.5)	
					Medication usage 8 (14.3)	13	Opioids	4 (30.8)	
							General analgesia	3 (23.1)	
							NSAIDs	3 (23.1)	
Clinician-reported outcomes	44 (47.8)	2 (2.1)	68	1.5 (0.8)	Post-operative complications 31 (70.5)	34	Unspecified (frequency only)	30 (88.2)	19
							Delirium incidence	3 (8.8)	
							NSQIP	1 (2.9)	
					Disease-specific assessments 14 (31.8)	17	KSS (knee/function only)	5 (29.4)	
							Harris Hip Score	5 (29.4)	
							HSS Knee Score/MDP^e^	2 (11.8)	
					Functional independence 12 (27.3)	12	Barthel index of ADLs	3 (25.0)	
							Days to independent walking	3 (25.0)	
							ILOA	3 (25.0)	
Biomarker outcomes	7 (7.6)	1 (1.1)	34	4.6 (3.3)	Lipid markers 4 (57.1)	16	Cholesterol	4 (25.0)	21
							HDL	3 (18.8)	
							LDL/triglycerides	3 (18.8)	
					Glucose markers 3 (42.9)	6	HbA1c	2 (33.3)	
							FBG	1 (16.7)	
							Random glucose	1 (16.7)	
					Inflammatory markers 3 (42.9)	6	CRP	3 (50.0)	
							IL-6	2 (33.3)	
							TNFα	1 (16.7)	

BMI: body mass index; CRP: C-reactive protein; EQ-5D: EuroQuol-5 Dimension; ER: external rotation; FBG: fasting blood glucose; HbA1c: glycated haemoglobin; HDL/LDL: high-density lipoprotein/low-density lipoprotein; HOOS: Hip Disability and Osteoarthritis Outcome Score; HSS: hospital for special surgery; ICECAP: ICEpop CAPability Measure; ILOA: Iowa Level of Assistance Scale; IL-6: interleukin-6; ISPOR: International Society for Pharmacoeconomics and Outcomes Research; KOOS: Knee Disability and Osteoarthritis Outcome Score; KSS: Knee Society Score; OHS: Oxford Hip Score; OKS: Oxford Knee Score; MDP: Merle d’Aubigne and Postel score; NSAIDs: Non-Steroidal Anti-Inflammatory Drugs; NSQIP: National Surgical Quality Improvement Program; SF-36/SF-12: 36-item or 12-item Short Form Health Survey; TNFα: tumour necrosis factor alpha; WOMAC: Western Ontario and McMaster Universities Osteoarthritis Index.

a Individual trials could collect multiple assessments within a given domain.

b Number of trials collecting at least one outcome within a concept of interest (denominator is the number of trials collecting at least one outcome in each domain).

c Individual trials could use multiple assessment tools within a concept of interest.

d Where two or more outcome assessment tools were equally as common in a given concept of interest, they are both presented separated by a forward slash (denominator is the total times a concept of interest was reported across trials).

e Whilst these outcome assessments have patient-reported components, it was specifically stated that a clinician completed these assessments in the respective studies, or that only a clinician-reported component of the assessment tool was collected.^76,95,105,116^

A total of 36 concepts of interest were identified, and 219 unique assessment tools were used. No single tool was reported in over 50% of trials. See eFigures 1–5 in the Supplementary Materials for all concepts of interest and assessment tools identified, classified by International Society for Pharmacoeconomics and Outcomes Research outcome assessment domain. Table 1 lists the most common concepts and tools used.

Patient-reported outcomes was the most commonly collected domain*,* reported a total of 286 times across 85 trials and had the most unique tools used across all domains (Table 1 and eFigure 1). The most frequently reported concepts of interest were disease-specific assessments (*n* = 70/286, 24%), pain (*n* = 49/286, 17%) and health-related quality of life (*n* = 48/286, 17%).

Performance-based outcomes was the second-most frequently collected domain, collected 225 times across 61 trials, and encompassing seven concepts of interest (eFigure 2). Functional tests (*n* = 65/225, 29%), assessed using six different tools, and muscle strength (*n* = 57/225, 25%), assessed using ten different tools/methods, were the most common concepts of interest.

Observer-reported outcomes and clinician-reported outcomes were reported at least once in 60% (*n* = 55/92) and 48% (*n* = 44/92) of trials, respectively. The most common observer-reported concept of interest was healthcare utilisation (*n* = 79/145, 54%), which was assessed using eight different tools, of which length of stay (*n* = 37/79, 47%) and decision to delay surgery (*n* = 14/79, 18%) were the most frequently utilised (Table 1 and eFigure 3). Post-operative complications was the most common concept of interest in clinician-reported outcomes (*n* = 34/68, 50%) (Table 1 and eFigure 4), which was typically measured as number of general medical complications (e.g. wound infections), and surgery specific complications (e.g. reduced range of movement). Only one study used a specific tool, National Surgical Quality Improvement Program, to report complications.

Only seven trials (8%) collected a biomarker outcome, which was the least frequently collected outcome domain, and included five concepts of interest (eFigure 5).

Seventy-two trials (78%) collected outcomes across at least three International Society for Pharmacoeconomics and Outcomes Research outcome assessment domains, seven trials (8%) collected outcomes in one domain only, and three trials (3%) collected outcomes in all five domains. The most common domains collected together were observer-reported and patient-reported outcomes (Table 2).

**Table 2.** Frequency of international society for pharmacoeconomics and outcomes research outcome domains collected together in trials, *n* (%).

ISPOR outcome domains	Observer-reported	Clinician-reported	Patient-reported	Biomarker
Performance-based	62 (67.4)	37 (40.2)	58 (63.0)	5 (5.4)
Observer-reported	x	46 (50.0)	76 (82.6)	7 (7.6)
Clinician-reported	x	x	42 (45.7)	4 (4.3)
Patient-reported	x	x	x	6 (6.5)

ISPOR: International Society for Pharmacoeconomics and Outcomes Research.

### Alignment of outcome reporting with Outcome Measures in Rheumatology Clinical Trials-Osteoarthritis Research Society International core domain set for total joint arthroplasty

**Figure 2.**
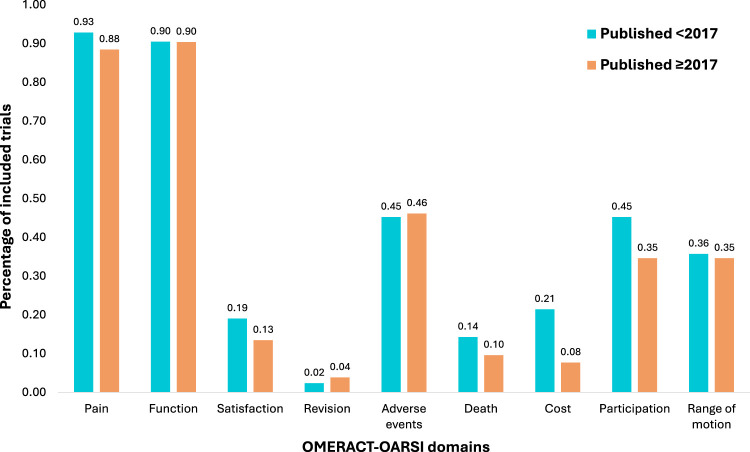
Analysis of changes in the percentage of individual outcome domains collected pre- and post-publication of the Outcome Measures in Rheumatology Clinical Trials-Osteoarthritis Research Society International core outcome domain set for total joint arthroplasty. This figure illustrates the percentage of trials included in the scoping review reporting at least one outcome measure within each of the domains of the core domain set, stratified by trials conducted pre-publication of the core domain set in 2017, and trials conducted post-release of the core domain set. OMERACT-OARSI: Outcome Measures in Rheumatology-Osteoarthritis Research Society International.

Overall, there was poor alignment of trial outcome reporting with the 2017 Outcome Measures in Rheumatology Clinical Trials-Osteoarthritis Research Society International core domain set. For trials published prior to its release in January 2017, there was an average of 41% alignment between outcome domains reported and those outlined in the core domain set. Trials initiated after the core domain set publication reported 37% of defined domains. When looking at the specific domains of the set, pain and function were most frequently collected, and this was the case both pre- and post-release of the core domain set (Figure 2). Satisfaction, cost, death and revision were the least frequently reported domains, which remained unchanged post-release of the core domain set.

### Timing of outcome collection

**Figure 3.**
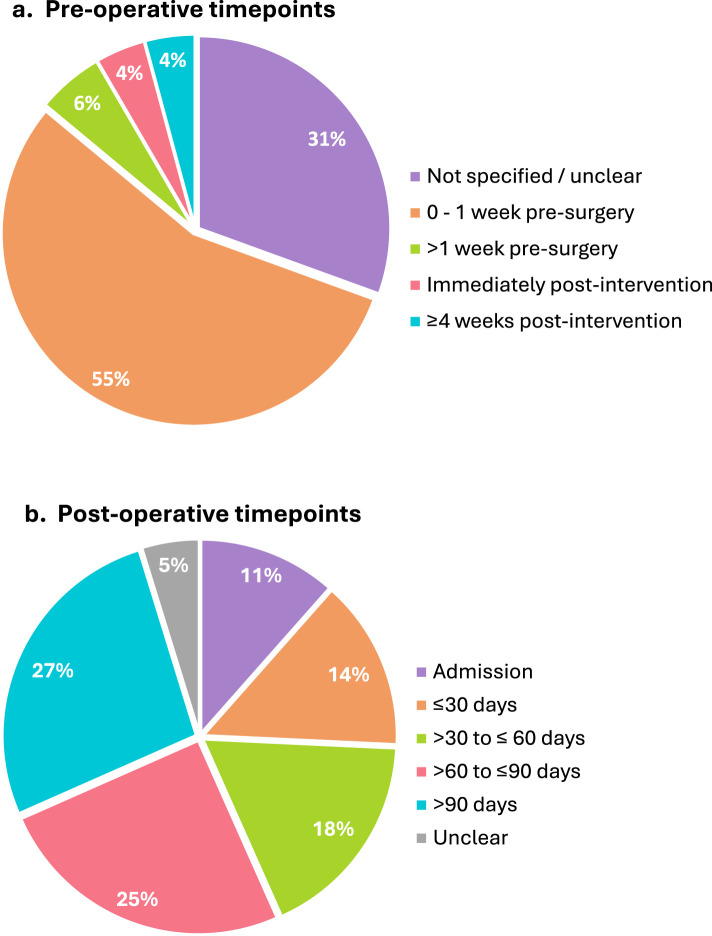
Timing of outcome measure collection in included trials.

Of the 92 trials, there were 94 papers with defined timepoints for follow-up outcome data collection (Skoffer et al.,^96^ and Skoffer et al.,^97^ and Villadesen et al.^110^ and Fernandes et al.,^46^ whilst reporting on the same samples, reported different timepoints). Seventy-two trials (77%) collected a pre-operative timepoint, 76 (81%) collected at least one post-operative timepoint, and 48 collected both a pre- and post-operative timepoint (51%). For trials that collected a pre-operative timepoint, almost a third did not specify the timing (Figure 3(a)). For trials that detailed when this timepoint was collected, there were two methods of reporting: relative to surgery and relative to intervention conclusion. Within 1 week pre-surgery was the most common timepoint described (*n* = 40, 56%) (Figure 3(a)). Only 13 trials (14%) disclosed the time between prehabilitation intervention conclusion and surgery, averaging 4 weeks.

(a) Pre-operative outcome measure timepoints and the percentage of trials collecting each respective time category. This refers to outcome measures that were collected before the operation but after the prehabilitation intervention.

(b) Post-operative outcome measure timepoints and the percentage collected in each respective time category across trials. Individual trials could collect multiple post-operative timepoints (e.g. ≤30 days and >60 to ≤90 days) and/or could collect multiple times within a time category (e.g. twice within the ≤30-day period). Admission was defined as the time from hospital admission until discharge from hospital care.

For post-operative outcome collection, 45 trials collected two or more follow-up timepoints. The most common post-operative assessment timepoint was >90 days (27%), followed by >60–90 days (25%) (Figure 3(b)). There were only 21 incidences (15%) where outcomes were collected within 30 days of surgery, and only two trials collected outcomes more than 1 year post-operatively.

### Prehabilitation intervention composition

Eighty-three trials (90%) delivered pre-operative-only interventions, while nine trials (10%) provided combined pre- and post-operative interventions.

**Figure 4.**
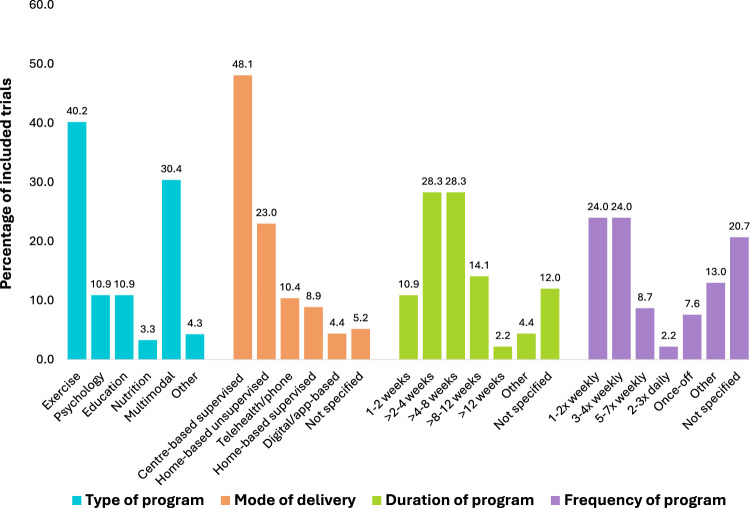
Prehabilitation program composition across included trials according to type of program, mode of delivery, duration of program and frequency of sessions.

Note: Mode of delivery data includes trials utilising one or a combination of delivery modes.

Sixty-four trials (70%) investigated unimodal prehabilitation, with exercise being the most common (*n* = 37, 40%), followed by psychological (*n* = 10, 11%), education (*n* = 10, 11%), nutrition (*n* = 3, 3%), and other modalities (*n* = 4, 4%) (Figure 4). ‘Other’ included respiratory (*n* = 1), neuromuscular stimulation (*n* = 1) and acupuncture interventions (*n* = 2). Twenty-eight trials (30%) explored multimodal prehabilitation, with exercise and education being the most commonly combined intervention types (*n* = 17/28, 61%). No trials combined exercise, nutrition and psychology components in one intervention.

Fifty-five trials (60%) used a single mode of prehabilitation delivery, most commonly supervised centre-based, while 37 trials (40%) used mixed modes, typically combining supervised centre-based and unsupervised home-based programs. Intervention duration was most frequently >2–4 weeks or >4–8 weeks (28%) and were delivered 1–2 times or 3–4 times (24%) per week (Figure 4).

### Outcomes according to prehabilitation type

**Figure 5.**
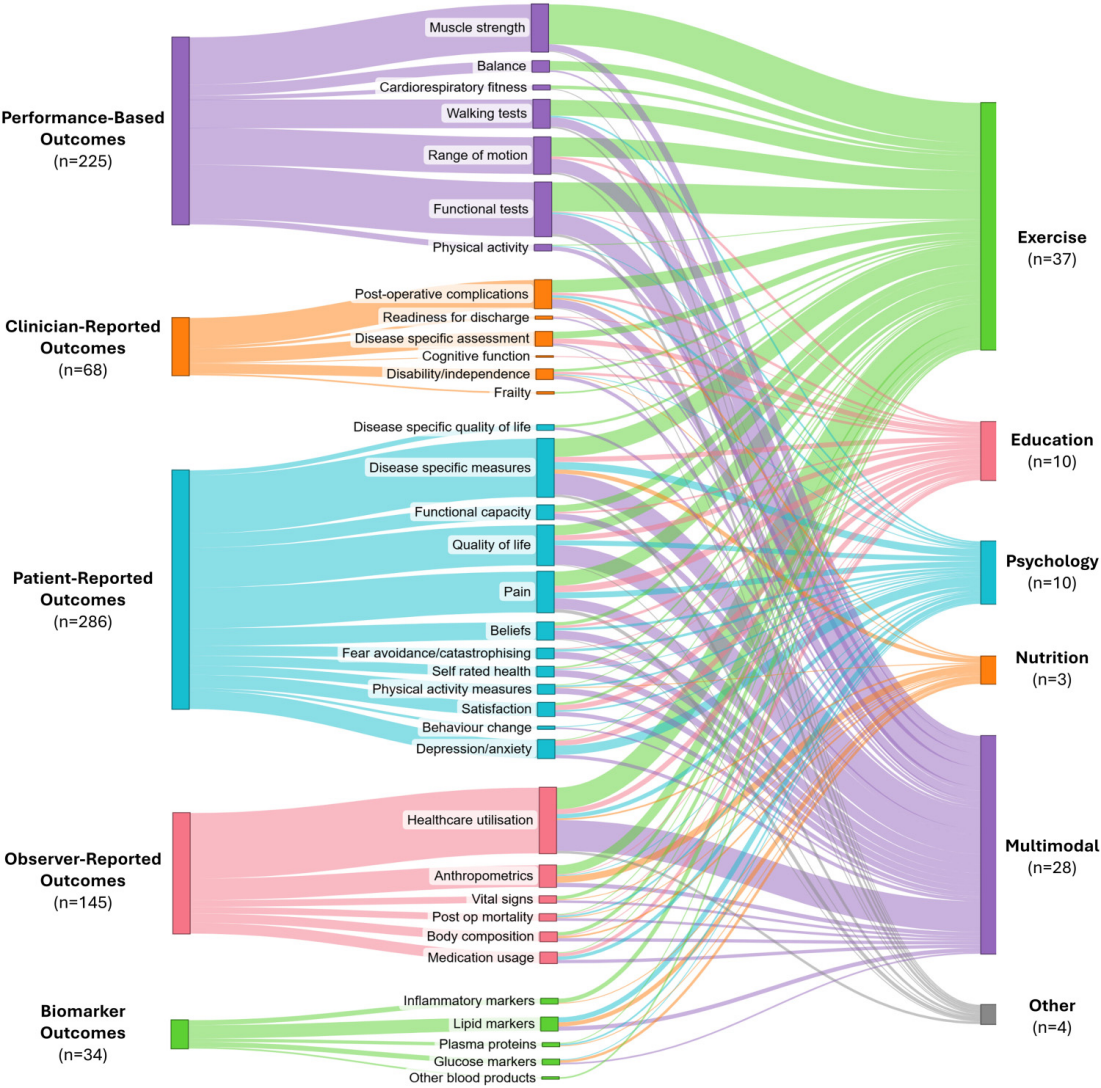
Sankey diagram describing the International Society for Pharmacoeconomics and Outcomes Research outcome assessment domains (left) and concepts of interest for outcome measures collected (middle) according to prehabilitation program type (right). The thickness of the connecting lines in this figure represents the proportion of trials reporting a particular outcome type.

Note: ‘Other’ interventions included respiratory (*n* = 1), neuromuscular stimulation (*n* = 1) and acupuncture (*n* = 2).

Figure 5 illustrates outcomes by prehabilitation type. For unimodal exercise prehabilitation, almost half of all outcomes collected were performance-based (48%), with muscle strength and functional tests being the most commonly collected concepts of interest. For unimodal psychological prehabilitation patient-reported was the most frequent outcome domain collected, with all trials collecting at least one patient-reported outcome, making up 60% of all outcomes reported by psychological prehabilitation trials. The most common concept of interest collected was depression and anxiety measures. For unimodal nutrition prehabilitation, of which there were only three trials, observer-reported and biomarker outcomes were the most prevalent domains collected, with anthropometrics and lipid markers being the most common concepts of interest within these domains, respectively. Education prehabilitation most frequently reported patient-reported outcomes, making up over 50% of all outcome measures reported for these trials (56%), with pain the most common concept of interest. Finally, all multimodal prehabilitation trials collected a patient-reported outcome (*n* = 28/28, 100%), with the most common concepts of interest being disease-specific measures (e.g. Oxford Hip Score) and health-related quality of life. All but two multimodal trials collected an observer-reported outcome (*n* = 26/28, 93%), with healthcare utilisation being the most common concept of interest.

### Process measures

Forty-eight trials (52%) reported adherence to the intervention, of which only ten defined acceptable levels of adherence *a priori.* Whilst most trials detailed how many prehabilitation sessions were planned in the intervention (*n* = 69, 75%), only 34 trials (37%) detailed how many sessions were delivered.

Cost analysis was reported in only 14 trials (15%) a total of 22 times (the same trial may have collected cost in multiple categories). Intervention and hospital-related costs were reported seven times each, community costs four times, and personal expenses were reported three times. Only one study performed a cost-utility analysis using quality adjusted life years. Costs were measured using diaries, questionnaires and data collected from national registries.

### ‘Non-outcome’ assessments

Several assessments were identified that were collected for purposes other than outcome measurement. To characterise participants at baseline, 20 trials (22%) used at least one graded comorbidity risk assessment, most commonly the American Society of Anesthesiologists physical status classification system, and 25 trials (27%) used at least one disease-specific risk assessment tool, with the Kellgren Lawrence Grading Scale being the most common.

Forty-two trials (46%) used at least one specific assessment tool with cut-off scores for inclusion/exclusion criteria. Fifteen trials (16%) used at least one assessment tool to select a higher risk cohort, with higher scores on the Pain Catastrophising Scale (*n* = 5) and body mass index (*n* = 4) the most frequently used tools. Four trials used frailty assessment tools to select a frailer cohort, each using a different assessment tool. Twenty-two trials (24%) used assessment tools to exclude higher risk participants, with American Society of Anesthesiologists physical status classification system (*n* = 8), body mass index (*n* = 9) and walking independence/distance (*n* = 5) the most common tools used. Five trials included a mix of high and low risk criteria; for example, Buvanendran et al.,^33^ included participants with a score of greater than 16 on the Pain Catastrophising Scale, but excluded participants with an American Society of Anesthesiologists physical status classification system of greater than 3. No trials used assessment tools to stratify the intervention delivered.

### Definition of prehabilitation

Prehabilitation was defined in only 22 trials (24%). Coding of the definitions mapped to six of the Template for Intervention Description and Replication domains, which became overarching themes:^22^ descriptor, why (goal), what (intervention type), how (delivery mode), who (participants targeted) and when (timing) (Table 3). Within each theme, the most common codes were identified (Table 3). Regarding the descriptor, the codes ‘rehabilitation prior to surgery’ and ‘preparation’ were the most common. The goal or intended outcome of prehabilitation was mentioned 28 times (across 15 definitions) and included eight different codes with the most common being to ‘improve or maintain functional capacity’ and ‘withstand stressor’ (*n* = 7 and *n* = 6, respectively). The intervention type was mentioned 24 times (across 16 definitions) and six codes were identified, with exercise/physical activity the most common (*n* = 16). Delivery mode and participants targeted were rarely mentioned (*n* = 3 and *n* = 1, respectively), and timing of prehabilitation was mentioned 16 times, with the predominant code being ‘pre-operative’ (*n* = 15).

**Table 3.** Identified themes and codes for prehabilitation definitions using directed content analysis.

TIDieR element/theme:	Codes	Count and frequency (*n* = 22 trials) *n* (%)^a^
Descriptor	Rehabilitation prior to surgery	5 (23)
	Preparation	3 (14)
	Presurgical intervention	1 (5)
	Structured exercise process	1 (5)
	*Total*	*10 (45)*
Why (goal)	Improve or maintain functional capacity	7 (32)
	Withstand stressor	6 (27)
	Improve post-operative outcomes	4 (18)
	Reduce complications/delayed recovery	4 (18)
	Enhance recovery	2 (9)
	Improve pre-operative outcomes	2 (9)
	Improve physiological reserve	2 (9)
	Optimise cardiorespiratory fitness	1 (6)
	*Total*	*28 (127)*
What (intervention type)	Exercise/ physical activity	16 (73)
	Education	2 (9)
	Psychology	2 (9)
	Nutrition	2 (9)
	Physiotherapy	1 (5)
	Occupational therapy	1 (5)
	*Total*	*24 (109)*
How (delivery mode)	Either unimodal or multimodal	2 (9)
	Multimodal	1 (5)
	*Total*	*3 (14)*
Who (participants targeted)	Frail	1 (5)
	*Total*	*1 (5)*
When (timing)	Pre-operative/pre-surgery	15 (88)
	Preceding treatment	1 (6)
	*Total*	*16 (73)*

TIDieR: template for intervention description and replication.

a Total times a code was reported across 22 definitions; studies may have reported multiple codes within one category.

## Discussion

This scoping review provides a comprehensive overview of reported outcome domains and assessment tools across timepoints in prehabilitation interventions for total hip and knee arthroplasty. It demonstrated considerable heterogeneity in concepts of interest and measurement tools, and poor alignment of outcome reporting with existing core domain sets. Patient-reported outcomes was the most frequently reported domain, and length of stay was the most frequent assessment tool. Exercise-only prehabilitation was the dominant intervention type, and prehabilitation was poorly defined.

Given surgery success is increasingly linked to patient-reported outcomes,^117^ it is unsurprising that most trials included a patient-reported outcome. However, high variability existed with 102 different tools utilised, echoing findings from previous studies,^13,118^ and complicating comparison across studies. Prior research demonstrates poor agreement between performance-based and patient-reported outcomes, thus future studies should include both.^119^ In this review, 63% of trials included both patient-reported and performance-based outcomes.

Length of stay, a common measure of prehabilitation efficacy,^13^ was the most frequently reported tool. With the introduction of enhanced recovery after surgery protocols, length of stay following total hip and knee arthroplasty has been significantly reduced.^120^ Length of stay can be influenced by many factors including institutional protocols, services supporting discharge and patient expectations.^121^ Potentially more meaningful measures of early post-operative recovery such as readiness for discharge^122^ or time to achieve functional milestones, were rarely reported, possibly due to the lack of validated tools to assess short-term post-operative function.^123^ Notably, 14 trials reported data on surgery deferral, though not as a predefined outcome. This may represent an unanticipated effect of prehabilitation in this cohort that warrants further investigation. Researchers and clinicians may consider monitoring surgery deferral as an outcome measure of prehabilitation programs.

Alignment of outcome measures with the 2017 Outcome Measures in Rheumatology Clinical Trials-Osteoarthritis Research Society International core domain set^12^ was also assessed. No notable change in reporting pre- and post-core domain set publication indicates poor uptake. Satisfaction, a core domain with validated tools available,^124^ was assessed in fewer than 20% of trials. Given that 10%–30% of patients report dissatisfaction post-total hip and knee arthroplasty^125–127^ satisfaction should be routinely assessed in prehabilitation trials and may be targeted pre-operatively through expectation setting.^128^ Cost analysis was also underreported with only 14 trials including cost data, consistent with broader prehabilitation literature.^13^ Economic evaluations are crucial for assessing prehabilitation feasibility and value and should include healthcare provider and societal perspectives.^129^ Standardised measures such as Quality Adjusted Life Years could support resource allocation and should be reported in future trials.

The poor alignment of outcome reporting with the 2017 core domain set suggests a need for improved dissemination and targeted education to enhance adoption. Unclear definitions and lack of consensus on tools may contribute to poor uptake. Alternatively, the current core domain set may not reflect prehabilitation goals, particularly in interventions targeting specific outcomes such as weight loss. Given differences between total hip and knee arthroplasty regarding post-surgical outcomes and prehabilitation effects,^9^ separate analyses and tailored outcome assessments may be needed. Until a core outcome set is established, outcome selection should consider psychometric properties, feasibility, cost and patient acceptability. The Oxford Knee/Hip Score, Western Ontario and McMaster Universities Osteoarthritis index, timed-up-and-go and six-minute walk test are among the most validated tools in this population.^130,131^

Almost 25% of included trials lacked a pre-operative outcome measure, limiting assessment of prehabilitation efficacy. Previous reviews have shown improvements in post-operative outcomes are greatest when prehabilitation is effective pre-operatively,^11^ highlighting the importance of pre-operative measures to evaluate effectiveness of the program. Furthermore, only 13 trials specified when the intervention concluded relative to surgery. If significant time has passed between prehabilitation concluding and surgery, any effect of the intervention may be lost. Future trials must report a post-intervention outcome timepoint, and length of time between prehabilitation conclusion and surgery. Reporting of adherence to the intervention, including *a priori* definitions of acceptable adherence levels, was poorly reported; a problem not isolated to orthopaedic prehabilitation trials.^132,133^ Without adherence data, it is difficult to discern whether negative outcomes relate to the intervention itself or implementation fidelity.^134^

Exercise-only prehabilitation was the most common program type. No trials combined exercise, nutrition and psychological components. A recent systematic review with network meta-analysis investigating prehabilitation across surgical populations reported that multimodal prehabilitation, in particular exercise and nutrition, provides moderate quality evidence to reduce complications and length of stay, and improve quality of life and physical recovery.^135^ Given the prevalence of obesity^136^ and psychological comorbidities^137^ in the hip and knee arthroplasty population, future trials should adopt targeted multimodal prehabilitation based upon comorbidity assessment, with corresponding outcomes. Lack of multimodality was also evident in the analysis of prehabilitation definitions. Only 24% of trials defined prehabilitation, most focusing on exercise only, underscoring the need for an orthopaedic-specific prehabilitation definition to guide program design and outcome measure selection.

The lack of risk screening assessments among included trials was notable. Nearly 25% of trials excluded high-risk participants, and only four included frail individuals. Risk factors such as frailty, obesity and psychological comorbidities predict poorer outcomes in total hip and knee arthroplasty,^138–140^ representing key targets for prehabilitation. Recent evidence recommends pre-operative interventions targeting prognostic indicators that increase risk of poor surgical outcomes in this cohort.^141^ Excluding high-risk individuals may dilute effects of prehabilitation. Future trials should prioritise inclusion of high-risk patients to evaluate prehabilitation in participants with varying risk profiles.

Strengths of this review include a rigorous librarian-assisted search strategy, dual-reviewer screening and extraction, and adherence to established scoping review methodology. Limitations include the restriction to English-language, post-2000, randomised controlled trials. While reflecting contemporary surgical practice and resource constraints, this may have resulted in an incomplete overview of reported outcomes, and language restrictions may under-represent trials which have been conducted in non-English speaking countries. Additionally, the alignment of outcome assessment tool administration with published consensus guidelines may represent an important variable, however, was considered beyond the scope of the present review. Consistent with scoping review guidelines,^14^ methodological quality of studies was not assessed.

This review is the first to comprehensively map outcome reporting in hip and knee arthroplasty prehabilitation, revealing substantial heterogeneity in what, how, and when outcomes are measured. Currently, the variation in outcome measure reporting is a barrier to prehabilitation implementation into clinical practice. To address this, there is a need to develop a core outcome set to provide guidance to researchers and clinicians on a standardised set of outcomes for use in clinical practice and research, facilitating improved data harmonisation and evidence synthesis.

## Clinical messages

- Evidence for prehabilitation effects on joint arthroplasty outcomes may be affected by heterogeneity in reported measures. 
- Meaningful, valid and feasible outcomes and assessment tools in this population need to be identified to form a core outcome set for future reporting. 

## Supplemental Material

sj-docx-1-cre-10.1177_02692155251378374 - Supplemental material for Outcome measures in prehabilitation interventions for total hip and knee arthroplasty: A scoping review Supplemental material, sj-docx-1-cre-10.1177_02692155251378374 for Outcome measures in prehabilitation interventions for total hip and knee arthroplasty: A scoping review by Nicola Burgess, Stefanie N Voelker, Belinda Phillips, Marnie Graco, Sue Berney, Linda Denehy and Lara Edbrooke in Clinical Rehabilitation
