# Supplementary material for: Outcome measures in prehabilitation interventions for total hip and knee arthroplasty: A scoping review
Source: Clin Rehabil. 2025 Sep 19;40(1):44–68. doi: 10.1177/02692155251378374 (PMC12722570; doi:10.1177/02692155251378374)
Supplement: sj-docx-1-cre-10.1177_02692155251378374 - Supplemental material for Outcome measures in prehabilitation interventions for total hip and knee arthroplasty: A scoping review [file sj-docx-1-cre-10.1177_02692155251378374.docx]

**SUPPLEMENTARY MATERIAL**

**Table of contents:**

**[eTable 1.](#eTable1)** [Search strategy](#eTable1)

[**eTable 2.** Definitions and examples according to the International Society for Pharmacoeconomics and Outcomes Research (ISPOR) framework](#eTable2)

**[eTable 3.](#eTable3)** [Excluded trials and reasons for exclusion](#eTable3)

**[eTable 4.](#eTable4)** [Summary of trial characteristics](#eTable4)

[**eTable 5.** Characteristics of included studies](#eTable5)

[**eFigure 1.** Patient-reported Outcome Measurement Tools](#eFigure1)

[**eFigure 2.** Performance-based Outcome Measurement Tools.](#eFigure2)

[**eFigure 3.** Observer-reported Outcome Measurement Tools](#eFigure3)

[**eFigure 4.** Clinician-reported Outcome Measurement Tools](#eFigure4)

[**eFigure 5.** Biomarker Outcome Measurement Tools](#eFigure5)

[References for included trials](#References)

**eTable 1.** Search strategy

| **Medline:** search run on the 9^th^ of May 2024, and re-run 15^th^ of May 2025 | |
| --- | --- |
| 1. | Knee Prosthesis/ or Arthroplasty, Replacement, Knee/ or Osteoarthritis, Knee/su [Surgery] |
| 2. | Hip Prosthesis/ or Arthroplasty, Replacement, Hip/ or Osteoarthritis, Hip/su [Surgery] |
| 3. | hip osteoarthritis.ti,ab,kf. |
| 4. | knee osteoarthritis.ti,ab,kf. |
| 5. | (knee adj3 (replace* or arthroplast* or prosthes* or total)).ti,ab,kf. |
| 6. | (hip adj3 (replace* or arthroplast* or prosthes* or total)).ti,ab,kf. |
| 7. | or/1-6 |
| 8. | Pre-operative Care/ or Pre-operative Period/ |
| 9. | (pre-operat* or preoperat* or pre surgery or pre-surgery or before surgery).ti,ab,kf. |
| 10. | (before adj1 (TKA or THA or total hip replacement or total knee replacement or total hip arthroplasty or total knee arthroplasty or total joint replacement or total joint arthroplasty)).ti,ab,kf. |
| 11. | ((prepar* or prior) adj2 (TKA or THA or total hip replacement or total knee replacement or total hip arthroplasty or total knee arthroplasty or total joint replacement or total joint arthroplasty)).ti,ab,kf. |
| 12. | or/8-11 |
| 13. | Pre-operative Exercise/ or exercise/ or exercise therapy/ or "Physical Education and Training"/ or Physical Therapy Modalities/ or physical fitness/ or cardiorespiratory fitness/ |
| 14. | (prehab* or pre-hab* or pre-operative exercise or pre-operative exercise).ti,ab,kf. |
| 15. | (exercise* or physical activit* or physical therap* or physical fitness or physiotherap*).ti,ab,kf. |
| 16. | Blood flow restriction therapy/ or endurance training/ or plyometric exercise/ or resistance training/ or High-Intensity Interval Training/ or proprioception/ or postural balance/ or core stability/ or electric stimulation therapy/ |
| 17. | ((blood flow restrict* or aerobic or endurance or plyometric* or strength or resistance or interval or high-intensity interval or high-intensity intermittent or proprioception or balance or postural sway or posture or core or core stability or neuromuscular electrical stimulation) adj1 (training or exercise or intervention or therapy)).ti,ab,kf. |
| 18. | Patient Education as Topic/ or health education/ or health literacy/ |
| 19. | (patient education or patient information or health education or health information or health literacy).ti,ab,kf. |
| 20. | Chronic Pain/ or Pain Management/ or Self-Management/ or Self Care/ |
| 21. | Cognitive psychology/ or stress, psychological/ or resilience, psychological/ or cognitive behavioral therapy/ or mindfulness/ |
| 22. | (chronic pain or pain education or pain management or self management or self-management).ti,ab,kf. |
| 23. | (cognitive therapy or cognitive behavio?ral or cognitive psychology or mindfulness or stress reduction or stress management or meditation or resilience).ti,ab,kf. |
| 24. | Nutrition therapy/ or diet therapy/ or nutritional support/ or dietary supplements/ or weight loss/ |
| 25. | (weight management or weight loss or nutrition* therapy or nutrition* support or diet* therapy or protein supplement*).ti,ab,kf. |
| 26. | "Activities of daily living"/ or occupational therapy/ |
| 27. | (occupational therapy or activities of daily living).ti,ab,kf. |
| 28. | Rehabilitation/ or telerehabilitation/ |
| 29. | (rehabilitation or tele-rehabilitation or telerehabilitation).ti,ab,kf. |
| 30. | or/13-29 |
| 31. | 7 and 12 and 30 |
| 32. | randomized controlled trial.pt. |
| 33. | controlled clinical trial.pt. |
| 34. | randomized.ab. |
| 35. | placebo.ab. |
| 36. | drug therapy.fs. |
| 37. | randomly.ab. |
| 38. | trial.ab. |
| 39. | groups.ab. |
| 40. | or/32-39 |
| 41. | exp animals/ not humans.sh. |
| 42. | 40 not 41 |
| 43. | 31 and 42 |
| 44. | limit 43 to yr="2000-current" |

*The search strategy, including all identified keywords and index terms, was adapted for the following databases: Embase, CINAHL, Web of Science, and Cochrane

**eTable 2**. Definitions and examples according to the International Society for Pharmacoeconomics and Outcomes Research (ISPOR) framework.

| **ISPOR terminology** | **Definition** | **Example** |
| --- | --- | --- |
| **Performance based outcome** | Involves a patient performing a task, but requires no rater perspective or clinical judgement to quantify performance | Timed up and go test to measure physical function |
| **Clinician reported outcome** | Requires the rater to have appropriate training, applying professional expertise or judgement to the observation | Iowa level of assistance scale to assess independence status |
| **Patient reported outcome** | Involves the patient as the rater and relies on the patient’s direct response without further interpretation from a clinician or observer. May be collected in varying formats (e.g., Interviews, paper, web-based) | 36-item short form survey to measure health-related quality of life |
| **Observer reported outcome** | Recorded by an observer (not the patient) who does not require specialised professional training | Hospital length of stay collected from the medical record |
| **Biomarker outcome** | A biochemical measure physically present in bodily fluids, subject to little to no patient motivational or rater judgmental influence | Glycated hemoglobin (HbA1c) to assess glucose metabolism |
| **Concept of interest** | What the outcome assessment intends to measure; often a simplified form of a meaningful aspect of the patient’s health or disease state | Psychological health (concept of interest) can be measured using the Hospital Anxiety and Depression Scale (HADS) |
| **Outcome assessment** | The specific instrument or tool used to provide a rating or score (categorical or continuous) that represents the concept of interest, and is used to obtain a measurement in a study endpoint |  |

**eTable 3.** Excluded trials and reasons for exclusion

| **Reference** | **Title** | **Reason for exclusion** |
| --- | --- | --- |
| Alghadir et al., 2016 | Comparison of the effect of pre- and post-operative physical therapy versus post-operative physical therapy alone on pain and recovery of function after total knee arthroplasty | Intervention started <7 days pre-operatively |
| Al-Heizan et al., 2023 | The efficacy of a pre-operative occupational therapy educational session for Saudi patients undergoing a lower extremity joint replacement | Wrong study design – not an RCT |
| An et al., 2021 | Effects of pre-operative telerehabilitation on muscle strength, range of motion, and functional outcomes in candidates for total knee arthroplasty: A single-blind randomized controlled trial | Wrong patient population – included bilateral TKA |
| Antonio et al., 2023 | Improvement of quality of life after knee prosthesis | Protocol only |
| Aoki et al., 2009 | Home stretching exercise is effective for improving knee range of motion and gait in patients with knee osteoarthritis | Wrong patient population – states “bilateral”, emailed authors x3 to clarify, no response |
| Aronsson et al., 2009 | A carbohydrate-rich drink shortly before surgery affected IGF-I bioavailability after a total hip replacement. A double-blind placebo controlled study on 29 patients | Intervention started <7 days pre-operatively |
| Aytekin et al., 2019 | The effect of a 12 week prehabilitation program on pain and function for patients undergoing total knee arthroplasty: A prospective controlled study | Wrong study design – not an RCT |
| Baas et al., 2024 | The effect of pain neuroscience education on chronic postsurgical pain after total knee arthroplasty: a randomized controlled trial | Preoperative + postoperative intervention, no pre-operative/post prehabilitation intervention outcome measure timepoint |
| Baran et al., 2020 | The effect of video information on pre-operative anxiety levels in patients undergoing total knee replacement | Intervention started <7 days pre-operatively |
| Bay et al., 2018 | Psychological coaching for patients undergoing total knee replacements | Protocol only |
| Bean et al., 2023 | Cognitive behavioural pain management prior to total knee joint replacement: a feasibility trial | Published abstract from a conference. Not an original research article |
| Bergin et al., 2010 | Interim analysis of the pre-operative incentive spirometry education (POISE) intervention...ASPAN National Conference | Interim/secondary analysis |
| Biau et al., 2015 | Neither pre-operative education or a minimally invasive procedure have any influence on the recovery time after total hip replacement | Wrong patient population – included avascular necrosis |
| Bitterli et al., 2011 | Pre-surgical sensorimotor training for patients undergoing total hip replacement: a randomised controlled trial | Wrong patient population – included avascular necrosis |
| Blong et al., 2023 | Improving Patient Activation Measure® in lower limb arthroplasty: Quantitative assessment of patient activation following a pre‐operative patient education programme | Wrong study design – not an RCT |
| Briguglio et al., 2023 | Oral iron powder for prehabilitation in hip and knee arthroplasty: A randomized controlled trial to optimize hemoglobin concentration | Does not meet prehabilitation definition – medical optimisation |
| Briguglio et al., 2020 | Oral supplementation with sucrosomial ferric pyrophosphate plus L-ascorbic acid to ameliorate the martial status: A randomized controlled trial | Does not meet prehabilitation definition – medical optimisation |
| Brosky et al., 2011 | Effects of prehabilitation on early rehabilitation outcomes following total knee arthroplasty in patients with knee osteoarthritis | Published abstract from a conference. Not an original research article |
| Bruce-Brand et al., 2012 | Effects of home-based resistance training and neuromuscular electrical stimulation in knee osteoarthritis: a randomized controlled trial | Wrong patient population – not all patients were waitlisted for surgery |
| Calverley et al., 2023 | Comparing two resources on pre-surgical physical activity and motivation for patients with hip/knee osteoarthritis | Protocol only |
| Canfield et al., 2021 | Evaluating the success of perioperative self-guided meditation in reducing sleep disturbance after total knee arthroplasty | Wrong study design – not an RCT |
| Casaña et al., 2022 | Exercise with blood flow restriction in knee osteoarthritis | Protocol only |
| Chen et al., 2020 | Does pre-operative balance training reduce pain and improve joint function in patients after total knee arthroplasty? A randomized controlled trial | Full text not available – pre- print publication |
| Chen et al., 2021 | Cognitive behavioral therapy cannot relieve post-operative pain and improve joint function after total knee arthroplasty in patients aged 70 years and older | Intervention started <7 days pre-operatively |
| Choudri et al., 2018 | The value of video-assisted education in pre-operative services in patients undergoing total knee replacement with regards to informed consent and anxiety using a modified Hamilton scale | Published abstract from a conference. Not an original research article |
| Christiansen et al., 2024 | Optimising total knee arthroplasty rehabilitation with telehealth physical activity before behaviour change intervention: A randomised controlled trial | Does not meet prehabilitation definition – delivered post-operatively |
| Chughtai et al., 2019 | The role of prehabilitation with a telerehabilitation system prior to total knee arthroplasty | Wrong study design – not an RCT |
| Cooke et al., 2016 | Pre-operative self-efficacy education vs. usual care for patients undergoing joint replacement surgery: a pilot randomised controlled trial | Wrong patient population – indication for surgery not collected (authors contacted) |
| Coudeyre et al., 2012 | Evaluation of an educational program associated with exercises (EDEX) before total knee arthroplasty | Protocol only |
| Crowe et al., 2003 | Pre-arthroplasty rehabilitation is effective in reducing hospital stay | Wrong patient population – included bilateral surgery |
| Culliton et al., 2018 | Effect of an e-learning tool on expectations and satisfaction following total knee arthroplasty: A randomized controlled trial | Preoperative + postoperative intervention, no pre-operative/post prehabilitation intervention outcome measure timepoint |
| Cusma et al., 2023 | Decreasing post-operative narcotic usage following total knee arthroplasty requires more than simple education: A blinded randomized controlled trial | Does not meet prehabilitation definition – did not provide multiple elements of education (brochure only) |
| Czyzewska et al., 2014 | Effects of pre-operative physiotherapy in hip osteoarthritis patients awaiting total hip replacement | Wrong study design – not an RCT |
| Dayucos et al., 2019 | Creation and evaluation of a pre-operative education website for hip and knee replacement patients-A pilot study | Wrong study design – no detail regarding randomisation strategy used. Emailed authors x3, no response |
| DeLuis et al., 2009 | Randomized clinical trial between nutritional counselling and commercial hypocaloric diet in weight loss in obese patients with chronic arthropathy | Not available in English |
| DeMik et al., 2024 | Association between digitally provided education and 90-day return to sexual activity following total knee arthroplasty: A randomized controlled trial | Interim/secondary analysis |
| Devereaux et al., 2014 | Intensive medical weight loss program achieves significant rapid pre-operative weight loss in the obese population | Published abstract from a conference. Not an original research article |
| Dindo et al., 2018 | Acceptance and commitment therapy for prevention of chronic postsurgical pain and opioid use in at-risk veterans: A pilot randomized controlled study | Wrong patient population - Mixed surgical population including hip/knee, spine and shoulder surgery without subgrouping |
| Dreyer et al., 2013 | Essential amino acid supplementation in patients following total knee arthroplasty | Preoperative + postoperative intervention, no pre-operative/post prehabilitation intervention outcome measure timepoint |
| Dreyer et al., 2018 | Essential amino acid supplementation mitigates muscle atrophy after total knee arthroplasty: A randomized, double-blind, placebo-controlled trial | Preoperative + postoperative intervention, no pre-operative/post prehabilitation intervention outcome measure timepoint |
| Du et al., 2023 | Study on the effect of pain programmed care based on the concept of prehabilitation on the recovery of joint function and WHOQOL-BREF score in elderly patients after total hip arthroplasty | Intervention started <7 days pre-operatively - Unable to determine when intervention commenced relative to surgery. Authors emailed x3, no response |
| Duan et al., 2022 | Short-term perioperative cognitive therapy combined with rehabilitation exercise reduces the incidence of neurocognitive disorder in elderly patients: a randomized controlled trial | Preoperative + postoperative intervention, no pre-operative/post prehabilitation intervention outcome measure timepoint |
| Eschalier et al., 2012 | Evaluation of a pre operative education approach for patient undergoing total knee replacement | Published abstract from a conference. Not an original research article |
| Eschalier et al., 2017 | Randomized blinded trial of standardized written patient information before total knee arthroplasty | Does not meet prehabilitation definition – did not provide multiple elements of education (brochure only) |
| Faurschou et al., 2007 | Effects of patient training | Not available in English |
| Fernandes et al., 2015 | Supervised neuromuscular exercise prior to hip or knee replacement: Cost-utility analysis alongside a randomised controlled trial | Published abstract from a conference. Not an original research article |
| Flack et al., 2020 | Hip abductor muscle volume in people with hip osteoarthritis following a targeted prehabilitation programme | Published abstract from a conference. Not an original research article |
| Gandler et al., 2016 | A pilot study investigating dietetic weight loss interventions and 12 month functional outcomes of patients undergoing total joint replacement | Intervention started <7 days pre-operatively – confirmed via email (once-off preop session) |
| Geng et al., 2021 | A randomized controlled trial of psychological intervention to improve satisfaction for patients with depression undergoing TKA: A 2-year follow-up | Intervention started <7 days pre-operatively |
| Giesche et al., 2017 | Pre-operative training for artificial knee and hip replacements | Not available in English |
| Gill et al., 2009 | Land-based versus pool-based exercise for people awaiting joint replacement surgery of the hip or knee: results of a randomized controlled trial | Wrong patient population – included bilateral surgery |
| Girishan Prabhu et al., 2024 | Designing and developing a nature-based virtual reality with heart rate variability biofeedback for surgical anxiety and pain management: evidence from total knee arthroplasty patients | Intervention started <7 days pre-operatively |
| Gocen et al., 2004 | The effect of pre-operative physiotherapy and education on the outcome of total hip replacement: a prospective randomized controlled trial | Wrong patient population – Included development dysplasia of the hip, idiopathic avascular necrosis of the hip and hip fractures |
| Grottanelli et al., 2007 | The relevance to the pre-surgery information to the patient subjected to hip arthroplasty | Not available in English |
| Haebich et al., 2015 | The effect of a web-based pre-operative coaching program on anxiety, pain and functional milestone attainment after knee replacement | Protocol only |
| Hanley et al., 2022 | A single-session, pre-operative mindfulness-based intervention improved surgical patients’ pre-and post-operative outcomes by encouraging self-transcendent states: results from two randomised clinical trials | Published abstract from a conference. Not an original research article |
| Hanley et al., 2021 | To be mindful of the breath or pain: Comparing two brief pre-operative mindfulness techniques for total joint arthroplasty patients | Wrong patient population – emailed authors to confirm, diagnosis/surgical indication not collected |
| Hanley et al., 2021 | Brief pre-operative mind-body therapies for total joint arthroplasty patients: a randomized controlled trial | Wrong patient population – emailed authors to confirm, diagnosis/surgical indication not collected |
| Hansen et al., 2012 | Pre-operative physical optimization in fast-track hip and knee arthroplasty | Wrong study design – not an RCT |
| Hashizaki et al., 2023 | Effectiveness of a 3-week rehabilitation program combining muscle strengthening and endurance exercises prior to total knee arthroplasty | Wrong study design – not an RCT |
| Hazar et al., 2023 | The effect of whole-body vibration treatment before total knee arthroplasty | Protocol only |
| Hermann et al., 2013 | Pre-operative resistance training increases muscle function in patients diagnosed with hip osteoarthritis scheduled for total hip arthroplasty A- a randomized explorative trial | Published abstract from a conference. Not an original research article |
| Hermann et al., 2014 | Pre-operative effects of progressive explosive-type resistance training in patients with osteoarthritis scheduled for total hip arthroplasty-a prospective randomized clinical trial | Published abstract from a conference. Not an original research article |
| Ho et al., 2022 | The effects of a patient-specific integrated education program on pain, perioperative anxiety, and functional recovery following total knee replacement | Wrong study design – not an RCT |
| Holsgaard-Larsen et al., 2018 | Post-operative effects of progressive resistance training prior to total hip arthroplasty – one year outcome of a randomised controlled trial | Published abstract from a conference. Not an original research article |
| Hoshi et al., 2016 | The effects of pre-operative rehabilitation on clinical outcomes after knee arthroplasty: a randomized controlled trial | Protocol only – study complete but no published article available |
| Inacio et al., 2014 | The impact of pre-operative weight loss on incidence of surgical site infection and readmission rates after total joint arthroplasty | Wrong study design – not an RCT |
| Iskender et al., 2020 | Effect of pre-operative in-bed exercises and mobilization training on post-operative anxiety and mobilization level | Intervention started <7 days pre-operatively |
| Jirakulsawat et al., 2024 | Assessment on knowledge and satisfaction level of delirium video for education in geriatric patients undergoing elective noncardiac surgery | Wrong patient population – included other surgical types, results not stratified by surgical type |
| Johansson et al., 2007 | Empowering orthopaedic patients through preadmission education: results from a clinical study | Wrong patient population – no mention of osteoarthritis. Emailed authors x3, no response |
| Jones et al., 2011 | Pre-operative patient education reduces length of stay after knee joint arthroplasty | Wrong study design – not an RCT |
| Jorgensen et al., 2024 | Is pre-operative blood flow restriction exercise superior to pre-operative usual care treatment before total knee arthroplasty on post-operative sit-to-stand function 3 months post-operatively: A randomized controlled trial | Published abstract from a conference. Not an original research article |
| Jorgensen et al., 2024 | Pre-operative low-load blood flow restricted exercise induce persistent gains in knee extensor muscle strength 3 months after total knee replacement surgery: Secondary analysis of a randomized controlled trial | Interim/secondary analysis |
| Karihtala et al., 2015 | Effects of pre-operative group-based aquatic training on health related quality of life in persons with late stage knee osteoarthritis | Published abstract from a conference. Not an original research article |
| Kennedy et al., 2014 | A pilot randomized control trial of aerobic cycling before total knee arthroplasty | Published abstract from a conference. Not an original research article |
| Kosek et al., 2013 | Increased pain sensitivity but normal function of exercise induced analgesia in hip and knee osteoarthritis--treatment effects of neuromuscular exercise and total joint replacement | Wrong study design – not an RCT |
| Kubo et al., 2020 | Effects of pre-operative low-intensity training with slow movement on early quadriceps weakness after total knee arthroplasty in patients with knee osteoarthritis: a retrospective propensity score-matched study | Wrong study design – not an RCT |
| Kubo et al., 2019 | Efficacy of 4 weeks pre-operative exercise with blood flow restriction on ischemia reperfusion injury after total knee arthroplasty | Protocol only |
| Laperche et al., 2022 | Obesity and total joint arthroplasty: Does weight loss in the pre-operative period improve perioperative outcomes? | Wrong study design – not an RCT |
| Lewis et al., 2002 | Patient knowledge, behavior, and satisfaction with the use of a pre-operative DVD | Wrong patient population – no mention of osteoarthritis, authors emailed x3, no response |
| Lia et al., 2025 | The effect of perioperative cognitive training on postoperative delirium in older patients undergoing total hip and knee arthroplasty: a prospective randomised trial | Intervention started <7 days pre-operatively |
| Liljensoe et al., 2015 | Weight loss intervention before total knee arthroplasty-feasibility and safety | Published abstract from a conference. Not an original research article |
| Liljensoe et al., 2014 | Weight loss intervention before total knee replacement. A safety study | Published abstract from a conference. Not an original research article |
| Lin et al., 2018 | Effects of nurse-led lower extremity strength training on knee function recovery in patients who underwent total knee replacement | Intervention started <7 days pre-operatively |
| Lin et al., 2018 | The effect of enhanced pre-operative education about continuous femoral blocks on analgesic effect and rehabilitation for patients undergoing total knee arthroplasty | Protocol only |
| Liu et al., 2024 | Effectiveness of a multimodal analgesia protocol in the perioperative period of knee replacement surgery in men | Does not meet prehabilitation definition |
| Ljunggren et al., 2012 | Oral nutrition or water loading before hip replacement surgery; a randomized clinical trial | Intervention started <7 days pre-operatively |
| Lu et al., 2023 | Pre- and post-operative education and health-related quality of life for patients with hip/knee replacement and hip fracture | Wrong study design – not an RCT |
| Lyp et al., 2016 | A water rehabilitation program in patients with hip osteoarthritis before and after total hip replacement | Wrong patient population - 2 groups had already received THA, 2 groups had osteoarthritis but not on a waiting list so not prehabilitation |
| Macchiaroli et al., 2012 | Relationships between pre-operative education, patient self-efficacy, patient anxiety, body mass index, and patient satisfaction of geriatric patients receiving primary total hip or total knee arthroplasty | Not original research article - dissertation |
| Maheu et al., 2024 | The efficacy of pre-operative video-based opioid counseling on post-operative opioid consumption after total knee arthroplasty: A prospective randomized controlled trial | Wrong patient population - no inclusion/exclusion regarding indication for TKA (authors emailed) |
| Mancuso et al., 2008 | Randomized trials to modify patients' pre-operative expectations of hip and knee arthroplasties | Wrong patient population – includes bilateral surgery |
| March et al., 2018 | A pilot study of a psychologically informed physiotherapy for people awaiting knee replacement surgery | Protocol only |
| Mat Eil Ismail et al., 2016 | Pre-operative physiotherapy and short-term functional outcomes of primary total knee arthroplasty | Wrong study design - “Patient assignment into two groups was randomised according to the week of their surgery”. Not a true RCT |
| McDonald et al., 2001 | Testing a pre-operative pain management intervention for elders | Wrong patient population – included revision surgery |
| McHugh et al., 2011 | The role of prehabilitation on the outcome of total knee arthroplasty: A randomized control trial | Published abstract from a conference. Not an original research article |
| Memtsoudis et al., 2014 | Pain after total knee arthroplasty? A randomized, double-blind study trial | Does not meet prehabilitation definition – medical optimisation |
| Meyer et al., 2023 | The effect of pre-operative behavioral intervention on pain, anxiety, opioid use, and function in patients undergoing total knee arthroplasty: A randomized controlled study | Wrong study design - randomised based on the month of their total joint class. Not a true RCT |
| Meyerkort    et al., 2020 | Low carbohydrate diet and blood sugar control in joint replacement | Protocol only |
| Mikkelsen et al., 2014 | Effect of early supervised progressive resistance training compared to unsupervised home-based exercise after fast-track total hip replacement applied to patients with pre-operative functional limitations. A single-blinded randomised controlled trial | Does not meet prehabilitation definition – intervention delivered post-surgery |
| Mir-Maroofi et al., 2016 | Randomized controlled trial of comparison of the self-efficacy and pain control in the patients undergoing total knee arthroplasty with and without empowerment program | Protocol only |
| Mitchell et al., 2005 | Costs and effectiveness of pre- and post-operative home physiotherapy for total knee replacement: randomized controlled trial | Preoperative + postoperative intervention, no pre-operative/post prehabilitation intervention outcome measure timepoint |
| Moretti et al., 2012 | I-ONE therapy in patients undergoing total knee arthroplasty: a prospective, randomized and controlled study | Does not meet prehabilitation definition – intervention delivered post-surgery |
| Muyskens et al., 2019 | Cellular and morphological changes with EAA supplementation before and after total knee arthroplasty | Wrong patient population – no mention of osteoarthritis, authors emailed x3, no response |
| Ninomiya et al., 2023 | Effects of perioperative exercise therapy combined with nutritional supplementation on functional recovery after fast-track total hip arthroplasty | Preoperative + postoperative intervention, no pre-operative/post prehabilitation intervention outcome measure timepoint |
| Nishizaki et al., 2015 | Effects of supplementation with a combination of β-hydroxy-β-methyl butyrate, L-arginine, and L-glutamine on post-operative recovery of quadriceps muscle strength after total knee arthroplasty | Intervention started <7 days pre-operatively |
| O'Connor et al., 2016 | YouTube videos to create a "virtual hospital experience" for hip and knee replacement Patients to decrease pre-operative anxiety: A randomized trial | Intervention started <7 days pre-operatively – unclear when intervention started, authors emailed x3, no response |
| Oestreich et al., 2015 | Prehabilitation for patients with osteoarthritis of the hip or knee waiting for a total joint replacement for hip or knee | Protocol only |
| Ohno et al., 2022 | Effect of 3-week pre-operative rehabilitation on pain and daily physical activities in patients with severe osteoarthritis undergoing total knee arthroplasty | Wrong study design – not an RCT |
| Oosting et al., 2015 | Feasibility of an intensive therapeutic exercise program for frail elderly prior to total hip arthroplasty: Two randomized pilot studies | Published abstract from a conference. Not an original research article |
| Pacheco-Brousseau et al., 2022 | Feasibility of a pre-operative strengthening exercise program on post-operative function in patients undergoing hip or knee arthroplasty: a pilot randomized controlled trial | No preoperative or postoperative efficacy outcome measures reported |
| Pellegrini et al., 2017 | Preliminary comparison of patient-centered weight loss programs starting before versus after knee replacement | Interim/secondary analysis |
| Pellegrini et al., 2018 | Comparison of a patient-centered weight loss program starting before versus after knee replacement: A pilot study | Wrong patient population - Included revision and bilateral surgery |
| Peretti et al., 2022 | Effects of tele-prehabilitation in patients waiting for knee replacement | Protocol only |
| Petersen et al., 2006 | Efficacy of multimodal optimization of mobilization and nutrition in patients undergoing hip replacement: a randomized clinical trial | Intervention started <7 days pre-operatively |
| Phillips et al., 2024 | A study to test the feasibility for adding a home-based Prehabilitation program prior to having total hip or knee surgery in older patients | Protocol only |
| Pinskiy et al., 2021 | The effect of a pre-operative physical therapy education program on short-term outcomes of patients undergoing elective total hip arthroplasty: A controlled prospective clinical trial | Wrong study design – not an RCT |
| Pour et al., 2007 | Minimally invasive hip arthroplasty: What role does patient preconditioning play? | Does not meet prehabilitation definition |
| Rezzan et al., 2015 | Efficacy of action observation pre-operative training in functional recovery after hip and knee prosthesis | Published abstract from a conference. Not an original research article |
| Riddle et al., 2019 | Pain coping skills training for patients who catastrophize about pain prior to knee arthroplasty: a multisite randomized clinical trial | Preoperative + postoperative intervention, no pre-operative/post prehabilitation intervention outcome measure timepoint |
| Rivard et al., 2003 | The efficacy of pre-operative home visits for total hip replacement clients | Wrong study design – not an RCT |
| Ródenas-Martínez et al., 2008 | Effectiveness of a pre-surgery rehabilitation program in total knee arthroplasty | Not available in English |
| Roy et al., 2005 | Creatine monohydrate supplementation does not improve functional recovery after total knee arthroplasty | Preoperative + postoperative intervention, no pre-operative/post prehabilitation intervention outcome measure timepoint |
| Saunders et al., 2017 | My Hip Journey : a virtual program for patients undergoing a total hip replacement | Protocol only |
| Saunders et al., 2021 | Comparing an eHealth program (My Hip Journey) with standard care for total hip arthroplasty: Randomized controlled trial | Preoperative + postoperative intervention, no pre-operative/post prehabilitation intervention outcome measure timepoint |
| Sekhar et al., 2023 | The role of pre-operative physical therapy in improving outcomes after total knee replacement: An observational study | Wrong study design – not an RCT |
| Shariaty et al., 2015 | Effect of educational program on pain intensity and range of motion in patients undergoing joint replacement | Protocol only |
| Shuo et al., 2020 | Effect of pre-operative balance training on post-operative recovery of total hip arthroplasty | Protocol only |
| Simpson et al., 2024 | A pre-operative package of care for osteoarthritis, consisting of weight loss, orthotics, rehabilitation, and topical and oral analgesia (OPPORTUNITY): a two-centre, open-label, randomised controlled feasibility trial | Wrong patient population – included 15-85 year olds |
| Simpson et al., 2024 | Is there a window of opportunity to effect positive health behaviour prior to surgery? A two centre open label randomised controlled feasibility trial of a pre-operative package of care for osteoarthritis, consisting of weight loss, orthotics, rehabilitation, topical and oral analgesia (OPPORTUNITY) | Published abstract from a conference. Not an original research article |
| Sjoling et al., 2003 | The impact of pre-operative information on state anxiety, post-operative pain and satisfaction with pain management | Wrong study design – not an RCT |
| Snowden et al., 2020 | Pre-operative behavioural intervention to reduce drinking before elective orthopaedic surgery: the PRE-OP BIRDS feasibility RCT | Does not meet prehabilitation definition – medical optimisation |
| Soni et al., 2010 | Severe knee osteoarthritis: A study of combined acupuncture and physiotherapy vs home exercise advice in patients awaiting total knee arthroplasty | Published abstract from a conference. Not an original research article |
| Sun et al., 2020 | Does cognitive behavioral education reduce pain and improve joint function in patients after total knee arthroplasty? A randomized controlled trial | Preoperative + postoperative intervention, no pre-operative/post prehabilitation intervention outcome measure timepoint |
| Suzer et al., 2024 | Effects of telerehabilitation-implemented core stability exercises on patient-reported and performance-based outcomes in total knee arthroplasty patients: randomised controlled trial | Does not meet prehabilitation definition – post-operative intervention |
| Szeverenyi et al., 2018 | Effects of therapeutic suggestions on the recovery of patients undergoing major orthopaedic surgery | Not available in English |
| Szilagyine Lakatos et al., 2022 | Cost-effective healthcare in rehabilitation: Physiotherapy for total endoprosthesis surgeries from prehabilitation to function restoration | Wrong study design – not an RCT |
| Thomas et al., 2018 | The effectiveness of pre-operative heat therapy and high-intensity interval training for prehabilitation in patients awaiting hip or knee arthroplasties | Protocol only |
| Tonnesen et al., 2023 | Two novel prehabilitation apps to help patients stop smoking and risky drinking prior to hip and knee arthroplasty | Does not meet prehabilitation definition – medical optimisation |
| Turan et al., 2025 | Preoperative patient education on opioid use and pain after surgery: A randomized trial | Wrong patient population – multiple surgery types included with no sub-analyses |
| Ueyama et al., 2020 | Perioperative essential amino acid supplementation suppresses rectus femoris muscle atrophy and accelerates early functional recovery following total knee arthroplasty: A prospective double-blind randmised controlled trial | Preoperative + postoperative intervention, no pre-operative/post prehabilitation intervention outcome measure timepoint |
| Ueyama et al., 2023 | Perioperative essential amino acid supplementation facilitates quadriceps muscle strength and volume recovery after TKA: A double-blinded randomized controlled trial | Preoperative + postoperative intervention, no pre-operative/post prehab intervention outcome measure timepoint |
| Valtonen et al., 2015 | Effects of pre-operative aquatic resistance training on knee pain, mobility limitation and muscle impairments in people with late-stage knee osteoarthritis | Published abstract from a conference. Not an original research article |
| VanDijk et al., 2015 | The effect of a pre-operative educational film on patients' post-operative pain in relation to their request for opioids | Wrong study design – not an RCT (quasi RCT) |
| Vasileidis et al., 2022 | Pre-operative rehabilitation in Greek patients undergoing total knee arthroplasty | Wrong study design – Quasi experimental |
| Villadsen et al., 2016 | Neuromuscular exercise prior to joint arthroplasty in patients with osteoarthritis of the hip or knee | Not original research article - dissertation |
| Villadsen et al., 2012 | Neuromuscular exercise (NEMEX-TJR) improves knee extension muscle power and chair stands ability in patients with severe knee osteoarthritis compared to controls: A randomized controlled trial | Published abstract from a conference. Not an original research article |
| Villadsen et al., 2011 | Neuromuscular exercise improves functional performance in patients with severe hip osteoarthritis | Published abstract from a conference. Not an original research article |
| Vosahlo et al., 2023 | Oral enzyme combination with bromelain, trypsin and the flavonoid rutoside reduces systemic inflammation and pain when used pre- and post-operatively in elective total hip replacement: a randomized exploratory placebo-controlled trial | Intervention started <7 days pre-operatively |
| Vukomanovic et al., 2008 | The effects of short-term pre-operative physical therapy and education on early functional recovery of patients younger than 70 undergoing total hip arthroplasty | Intervention started <7 days pre-operatively |
| Wang et al., 2024 | Influence of pre-rehabilitation training of quadriceps muscle strength on knee rehabilitation after total knee arthroplasty | Protocol only |
| Wang et al., 2020 | Effect of pre-operative balance training on post-operative recovery of unicompartmental knee arthroplasty | Protocol only |
| Wasim et al., 2024 | The effect of video-assisted learning on pre-operative knowledge and satisfaction for total knee arthroplasty surgery: a randomised-controlled study | Intervention started <7 days pre-operatively – unclear when intervention commenced. Emailed authors x3, no response |
| Weaver et al., 2003 | Comparison of two home care protocols for total joint replacement | Wrong patient population – includes bilateral TKA |
| Whale et al., 2025 | The role of health psychology in surgical prehabilitation: Insights from REST, a preoperative sleep intervention for total knee replacement patients | Interim/secondary analysis |
| Whitney et al., 2002 | Pre-operative physical activity, anesthesia, and analgesia: effects on early post-operative walking after total hip replacement | Interim/secondary analysis |
| Williamson et al., 2007 | Severe knee osteoarthritis: a randomized controlled trial of acupuncture, physiotherapy (supervised exercise) and standard management for patients awaiting knee replacement | Wrong patient population – includes unicompartmental and bilateral replacements |
| Wylde et al., 2014 | Effectiveness and cost-effectiveness of a group-based pain self-management intervention for patients undergoing total hip replacement: feasibility study for a randomized controlled trial | No preoperative or postoperative efficacy outcome measures reported |
| Xiaorong et al., 2023 | Development and implementation of a program for the prevention of chronic pre-operative pain among patients undergoing total knee arthroplasty | Protocol only |
| Yu et al., 2021 | Effect of psychological intervention care on the negative mood and hope level of elderly patients undergoing knee arthroplasty | Wrong patient population - Included rheumatoid arthritis and traumatic arthritis, <80% osteoarthritis |
| Zheng et al., 2022 | The effect of pre-operative rehabilitation training on the early recovery of joint function after artificial total knee arthroplasty and its effect evaluation | Intervention started <7 days pre-operatively – unclear when intervention commenced. Emailed authors x3, no response |

*Abbreviations: THA: Total Hip Arthroplasty; TKA: Total Knee Arthroplasty; RCT: Randomised Controlled Trial*

**eTable 4.** Summary of trial characteristics

| **Characteristics** | **Count and percentage**  **n (%)** |
| --- | --- |
| **Year of publication (n=94)*** |  |
| 2000-2005 | 9 (10.3) |
| 2006-2010 | 9 (10.3) |
| 2011-2015 | 18 (20.7) |
| 2016-2020 | 24 (27.6) |
| 2021-2025 | 34 (39.1) |
| **Trial type (n=92)*** |  |
| Randomised controlled trial | 69 (75) |
| Pilot/feasibility RCT | 23 (25) |
| **Location of trial (n=92)** |  |
| Europe | 40 (43.5) |
| United States | 14 (15.2) |
| United Kingdom | 13 (14.1) |
| Asia | 10 (10.9) |
| Australia | 6 (6.5) |
| Canada | 5 (5.4) |
| South America | 2 (2.2) |
| New Zealand | 1 (1.1) |
| South Africa | 1 (1.1) |
| **Trial population (n=92)** |  |
| TKA | 55 (59.8) |
| THA | 18 (19.6) |
| TKA & THA | 19 (20.7) |

Footnote: *94 papers reporting on 92 unique trials

*Abbreviations: THA: Total Hip Arthroplasty; TKA: Total Knee Arthroplasty; RCT: Randomised Controlled Trial*

**eTable 5.** Characteristics of included studies

| **Authors, ref** | **Year** | **Study type** | **Country** | **Surgical type** | **Age (years), mean (SD)** | **Sample size, gender (F)** | **Intervention** | **Duration/ frequency of intervention** | **Primary outcome*** | **Timepoints of outcome collection**** |
| --- | --- | --- | --- | --- | --- | --- | --- | --- | --- | --- |
| Ampachim et al., ^1^ | 2024 | RCT | Greece | THA | 66.3 (8.3) | 102, 72 F | Structured preoperative education and information program | 20 min sessions, one week pre-op, one day pre-op and postoperatively, before discharge | Not specified | Pre-op:1 week  Post-op: 4 weeks |
| Aunger et al., ^2^ | 2020 | Feasibility | England | THA & TKA | 73.1 (5.8) | 35, 20 F | Behavioural change intervention based on Self-Determination Theory- reduce sedentary time, motivational interviewing, behavioural goal setting | Up to 2 in person session and 3 phone calls | Feasibility measures | Pre-op: 1 week  Post-op: 6 weeks |
| Beaupre et al., ^3^ | 2004 | RCT | Canada | TKA | Intervention: 67 (7)  Control: 67 (6) | 131, 72 F | Strength exercises and education program | 4-week program, 3x weekly | WOMAC | Pre-op: unclear - “immediately pre-operatively"  Post-op: 3, 6, and 12 months |
| Berge et al., ^4^ | 2014 | RCT | United States | THA | Intervention: 71.6 (6)  Control: 71 (6.1) | 67, 27 F | Pain management program (group based with 9-10 participants) run by psychologist, occupational therapist and physiotherapist | 6-week program, 1-2x weekly | Not specified | Pre-op: 3 months post-intervention  Post-op: 12 months |
| Bergin et al., ^5^ | 2014 | RCT | United States | THA & TKA | 63.8 (8.7) | 140 (106 analysed), 61 F | Incentive spirometry training | 10 times every 2 hours while awake for 1 week before surgery | Inspiratory lung volume | Pre-op: nil  Post-op: Perioperative (until discharge) |
| Bertram et al., ^6^ | 2024 | Feasibility | England | TKA | Not specified | 57, 34 F | Tailored sleep assessment and behavioural intervention package | Unclear | Feasibility measures | Pre-op: 1 week  Post-op: 12 weeks |
| Birch et al., ^7^ | 2020 | RCT | Denmark | TKA | Intervention: 66 (9)  Control: 66 (10) | 60, 40 F | One-on-one patient education based on cognitive behavioural therapy | 7 sessions, 3 sessions preoperatively and 4 sessions postoperatively | VAS pain during activity | Pre-op: nil  Post-op: 12 and 52 weeks |
| Blasco et al., ^8^ | 2020 | RCT | Spain | TKA | 72.1 (7.6) | 86, 53 F | Group 1: outpatient intervention supervised focused on lower limb strengthening and balance.  Group 2: same program at home (1x supervised session at hospital for training purposes) | 4-week program, 2-3x weekly | Berg Balance Scale and KOOS- Function | Pre-op: 1 week  Post-op: 10-14 days and 6 weeks post-op |
| Brown et al., ^9^ | 2014 | RCT | United States | TKA | Prehab: 60 (8.3)  Control:  67 (9.5) | 37, 22 F | 5 component exercise program based on Social Cognitive Theory - warm-up, resistance, flexibility, step training and cool down | 3 times per week for 8 weeks | Self-efficacy for exercise and outcome expectation of exercise scales | Pre-op: 1 week Post-op: 1 and 2 weeks post-op |
| Brown et al., ^10^ | 2012 | Pilot study | United States | TKA | Not specified | 32, 18 analysed, M:F not specified | 5 component exercise program - warm-up, resistance, flexibility, step training and cool down | 3x weekly, once at home and twice in-centre, for 8 weeks | SF-36 | Pre-op: nil  Post-op: 3 months post op |
| Buvanendran et al., ^11^ | 2021 | RCT | United States | TKA | Phase I:  8-week telehealth: 58 (range 54-66) 4-week telehealth: 58 (range 52-62) 4-week in-person: 62 (range 60-69)  Control: 65 (range 50-74)    Phase II: Intervention: 66 (range 58-70)  Control:  62 (range 55-73) | Phase I: 80, 46 F  Phase II: 80, 51 F | Telehealth and in-person cognitive behavioural therapy (CBT) | Phase I: 8-week telehealth CBT, 4-week telehealth CBT, 4-week in-person CBT    Phase II: 4-week telehealth CBT | Phase I: pain catastrophising scale    Phase II: WOMAC | Phase I:  Pre-op: 1 week Post-op: nil    Phase II: Pre-op: 1 week Post-op:  3 months |
| Calatayud et al., ^12^ | 2017 | RCT | Denmark | TKA  TKA | 66.7 (3.9) | 50, analysed 44, 37 F | High intensity preoperative resistance training | 8-week training programme 3 days per week | WOMAC | Pre-op: unclear (“after 8 weeks of training and prior to surgery”)  Post-op: 1 month and 3 months |
| Casana et al., ^13^ | 2019 |  |  |  |  |  |  |  | Romberg test with eyes open and closed |  |
| Cannata et al., ^14^ | 2021 | RCT | Italy | THA | 73 (6) | 65, 36 F | Weight loss diet (fiber-enriched high carbohydrate diet) | 3 months | Oxford Hip Score, HOOS and WOMAC | Pre-op: unclear - “at follow-up after 3 months”  Post-op: nil |
| Cavill et al., ^15^ | 2016 | Pilot | Australia | TKA & THA | Intervention: 65.3 (9.6)  Control: 67.0 (9.4) | 64, 33 F | Circuit-based exercise program (group-based) | One-hour twice-weekly sessions for at least three and a maximum of four weeks | EQ-5D-3L and the patient-specific functional scale | Post-op: 8 weeks |
| Chen et al., ^16^ | 2024 | RCT | China | THA | 63.2 (7.1) | 60 (in pre-op groups), 25 F | Progressive resistance training | 2-week program, 3x weekly | Isokinetic strength of hip flexion, extension, adduction, and abduction | Pre-op: 1 day  Post-op:1, 3, 6, and 12 months |
| Crotty et al., ^17^ | 2009 | RCT | Australia | THA & TKA | 67.5 (10.5) | 152, 92 F | Self-management support program (self-management course, individualised phone support, goal setting) | 6-week program | Health Education Intervention Questionnaire | 6 months after randomisation |
| dasNair et al., ^18^ | 2018 | Feasibility | England | TKA | 66.2 (9.3) | 51 (50 analysed), 23 F | Psychological intervention, based on CBT for anxiety, depression, and pain management | Up to 10 sessions | WOMAC | 4 and 6 months post randomisation |
| deLuis et al., ^19^ | 2012 | RCT | Spain | THA & TKA | 65.0 (8.5) | 40, 33 F | Diet I: Optisource  Diet II: dietary advice to restrict intake by 500 cal/day | 3 months | Weight loss | Pre-op: day prior to surgery  Post-op: unclear - “3 months post intervention” |
| Doiron-Cardin et al., ^20^ | 2020 | Pilot | Canada | THA & TKA | Telehealth: 69.9 (9.1)  In-person:  61.3 (8.1)  Control:  66.7 (9.2) | 34, 25 F | 2 groups: in person exercise-based prehabilitation versus telehealth prehabilitation | 12-week program | Lower Extremity Functional Scale | Pre-op: Unclear - “after 12 weeks”  Post-op: unclear |
| Dominguez-Navarro et al., ^21^ | 2021 | RCT | Spain | TKA | Control:  70.2 (5.6)  Strength: 70.8 (5.4)  Strength + balance: 70.4 (6.4) | 82, 41 F | 2 groups: strengthening exercises, or strengthening plus balance exercises | 4-week program, 12 sessions | Berg Balance Scale and KOOS ADL subscale | Pre-op: 1 week  Post-op: 2, 6 and 52 weeks |
| Dowsey et al., ^22^ | 2019 | RCT | Australia | THA & TKA | Intervention: 65.8 (9.4)  Control:   65.1 (9.2) | 127, 92 F | Mindfulness-based stress reduction program (group based) | 8-week program, 1x weekly sessions | WOMAC | Pre-op: N/A  Post-op: 3 and 12 months |
| Evgeniadis et al., ^23^ | 2008 | RCT | Greece | TKA | 68.76 (5.64) | 72 (53 analysed), 42 F | Home based exercise program focused on strengthening trunk and upper extremities | 3-week program, 3x weekly | SF-36 | Pre-op: 1 day Post-op: 3 days, day of discharge, 6, 10 and 14 weeks |
| Ferrara et al., ^24^ | 2008 | RCT | Italy | THA | Intervention: 63.82 (9.01)  Control: 63.08 (6.89) | 23, 14 F | Group and individual exercises | 4-week program, 5x weekly | Not specified | Pre-op: 1 day  Post-op: 15 days, 4 weeks, 3 months |
| Franz et al., ^25^ | 2022 | RCT | Germany | TKA | 63.5 (8.1) | 30, 12 F | Cycling-ergometer-based training +/- blood flow restriction | Twice per week over 6 weeks | Not specified | Pre-op: 3 weeks and 5 days  Post-op: 12 and 26 weeks |
| Gilbey et al., ^26^ | 2003 | RCT | Australia | THA | 65.16 (11.11) | 76 (68 analysed), 42 F | 30-minute aerobic and strength program followed by a 30-minute program of mobility and gait training in the hydrotherapy pool | 8-week program, 2 supervised clinic-based and two home-based exercise sessions each week | Not specified | Pre-op: 1 week  Post-op: 3, 12 and 24 weeks |
| Giraudet-LeQuintrec et al., ^27^ | 2003 | RCT | France | THA | Intervention: 62.7 (8.8) Control: 64.3 (9.5) | 100, 44 F | Group education session (collective multidisciplinary information session) | Once-off session | State-Trait Anxiety Inventory | Pre-op: 1 day  Post-op: 7 days |
| Granicher et al., ^28^ | 2024 | Pilot | Switzerland | TKA | 72.70 (5.95) | 20, 15 F | Supervised exercise and education program | 4-8-week program, 2x sessions weekly | Feasibility | Pre-op: “immediately pre-surgery  Post-op: 6 and 12 weeks |
| Granicher et al., ^29^ | 2020 | Pilot | Switzerland | TKA | 67.35 (7.44) | 20, 8 F | Mixed training (endurance, proprioceptive neuromuscular facilitation) and patient education | 4-week program | Stair climb test | Pre-op: "immediately before surgery”  Post-op: 12 weeks |
| Gstoettner et al., ^30^ | 2011 | RCT | Austria | TKA | Intervention: 72.8 (range 65–78)  Control: 66.9 (range 61–75) | 38, 30 F | Preoperative proprioceptive training | 6-week program, daily sessions | Not specified | Pre-op: 1 day  Post-op: 6 weeks |
| Haslam et al., ^31^ | 2001 | RCT | England | THA | Group A: 66 (range 39-77)  Group B: 68 (range 57-77) | 32, 21 F | Group A: Acupuncture  Group B: advice and exercises | Group A: 6 session  Group B: 3 sessions | Modified WOMAC | Pre-op: Immediately post-treatment, and 8 weeks post-treatment  Post-op: nil |
| Hermann et al., ^32^ | 2016 | RCT | Denmark | THA | 70.4 (7.6) | 80, 52 F | Explosive resistance training program, group training (up to 8 participants) | 10 weeks, 2x weekly | HOOS ADL function subscale | Pre-op: 1-7 days  Post-op: nil |
| Hoogeboom et al., ^33^ | 2010 | Pilot | Netherlands | THA | 76 (4) | 21, 14 F | Outpatient physio exercise program | 3-to-6-week program, 2x weekly | Feasibility | Pre-op: nil  Post-op: each hospitalised day until discharge |
| Huang et al., ^34^ | 2012 | RCT | Taiwan | TKA | 70.2 (7.3) | 243, 174 F | Thigh muscle strength training + educational program | 2–4-week program | Not specified | Pre-op: day 1 of admission  Post-op: day 1 and 5 |
| Huber et al., ^35^ | 2015 | RCT | Switzerland | TKA | Intervention: 68.8 (8.0)  Control: 71.9 (8.1) | 45, 21 F | Neuromuscular exercise program + knee school educational package | 4 to 12 weeks, 2x weekly | Chair stand test | Pre-op: 1 week  Post-op: 6, 12 and 52 weeks |
| Husted et al., ^36^ | 2022 | RCT | Denmark | TKA | 66.7 (9.9) | 140, 76 F | Home-based resistance exercise program | 3 groups - 2, 4 or 6 sessions per week for 12 weeks | Isometric knee extensor strength | Pre-op: unclear - “before surgery”  Post-op: hospital discharge and 12 weeks |
| Ibrahim et al., ^37^ | 2014 | Pilot | United States | THA & TKA | Intervention: 65.3 (9.6)  Control: 67 (9.4) | 64, 33 F | Group-based exercise and education | 3-4 weeks, 2x weekly | EQ-5D-5L and Patient Specific Functional Scale | Pre-op: nil  Post-op: 8 weeks |
| Jahic et al., ^38^ | 2018 | RCT | Bosnia and Herzegovina | TKA | Males: 59.0 (9.47)  Females: 59.7 (6.28) | 20, 14 F | Quadriceps strength, flexibility and resistance training | 3x daily for 6 weeks | Not specified | Pre-op: “just prior to surgery”  Post-op: perioperative, 3, 6, and 12 months |
| Jepson et al., ^39^ | 2016 | Feasibility | England | THA | 66 (10.8) | 44, 20 F | Pre-operative occupational therapy intervention (home visit) | Once-off session | Feasibility | Pre-op: nil  Post-op: 4, 12 and 26 weeks |
| Jintana et al., ^40^ | 2020 | RCT | Thailand | TKA | Aged between 60-69: 49  Aged between 70-79: 43 | 96 (92 analysed), 82 F | Preoperative quadriceps exercise and diet control program | 12-week program, 3x weekly | Not specified | Pre-op: week 2, 8, and 12 (unclear when in relation to surgery  Post-op: nil |
| Jorgensen et al., ^41^ | 2024 | RCT | Denmark | TKA | 66 (95% CI 64.9-67.7) | 86, 49 F | Blood flow restriction resistance training | 8-week program, 3x weekly | 30 second sit-to-stand | Pre-op: approx. 3 days  Post-op: 12 and 52 weeks |
| Kaynar et al., ^42^ | 2023 | RCT | United States | TKA | Not specified | 15, M:F not specified | Digital cognitive behavioural intervention | 4-week program | PROMIS emotional anxiety and depression short form 8a | Pre-op: unclear - “prior to surgery”  Post-op: nil |
| Khalid et al., ^43^ | 2024 | Feasibility | England | THA & TKA | 75 (range 65–87) | 64, 46 F | Home exercise program + protein supplement | 12-week program, daily exercise and supplement | Feasibility | Pre-op: unclear (12 weeks post randomisation)  Post-op: nil |
| Kim et al., ^44^ | 2021 | Pilot | United States | TKA | 67.2 (6.1) | 43, 19 F | Aquatic exercises | 4-to-8-week program, 3x weekly | 30-day NSQIP | Pre-op: within 1 week  Post-op: 4 weeks |
| Kubo et al., ^45^ | 2024 | RCT | Japan | TKA | 73 (IQR 70-75) | 22, 18 F | Resistance and aerobic exercise + electrotherapy with vs without blood flow restriction | 4-week program, 2-3x weekly | Quadriceps strength | Pre-op: 1 week, immediately pre-op  Post-op: 24 hours, 4 days, 4 weeks and 12 weeks post-op |
| Leal-Blanquet et al., ^46^ | 2013 | RCT | Spain | TKA | Intervention: 72.1 (7.4)  Control: 73.4 (6.5) | 92, 70 F | Standard information + additional medical information through audiovisual videodiscs | Once-off session | Not specified | Pre-op: 4 weeks (post-intervention)  Post-op: nil |
| Liljensoe et al., ^47^ | 2021 | RCT | Denmark | TKA | Intervention: 65 (46-81)  Control: 65 (46-85) | 77, 54 F | Low-energy liquid diet (810kcal/day)  and nutritional education held in group session (goal to reduce body weight by 5-10%) | 8-week program with daily supplements and weekly education sessions | SF-36 physical component score | Pre-op: within 1 week  Post-op: 8, 26 and 52 weeks |
| Lluch et al., ^48^ | 2018 | RCT | Spain | TKA | Biomedical education: 72.8 (5.6)  Neuroscience education: 67.7 (7.8) | 54 (44 analysed), 28 F | Pain neuroscience education with knee joint mobilisation vs. biomedical education with knee joint mobilisation | 4-week program, 1x weekly | Conditioned Pain Modulation | Pre-op: immediately post intervention and 1 month post intervention  Post-op: 3 months |
| Matassi et al., ^49^ | 2014 | RCT | Belgium | TKA | Intervention: 66 years (7.2)  Control: 67 years (7.7) | 122, 59 F | Flexibility and strength home-based exercise program | 6-week program, 5x weekly | Passive knee flexion | Pre-op: 1 day  Post-op: 6, 26, and 52 weeks |
| McGregor et al., ^50^ | 2004 | Pilot | England | THA | 71.9 (9.3) years | 35, 25 F | Preoperative rehabilitation advice reinforced by a patient information booklet | Once-off session | Not specified | Pre-op: admission to hospital  Post-op: before discharge, 3 months |
| McKay et al., ^51^ | 2012 | Pilot | Canada | TKA | Intervention: 63.5 (4.93)  Control: 60.58 (8.05) | 22, 13 F | Circuit of bilateral lower body exercises | 6-week program, 3x weekly | Isometric quadriceps strength | Pre-op: “immediately prior to surgery”  Post-op: 6 and 12 weeks |
| Medina-Garzon et al., ^52^ | 2019 | RCT | Colombia | TKA | Intervention: 76.32 (16.1)  Control: 73.7 (16.6) | 56, 26 F | Motivational interviewing to reduce preop anxiety | 3-week program | Amsterdam Preoperative Anxiety and Information Scale | Pre-op: 4 weeks post intervention  Post-op: nil |
| Nam et al., ^53^ | 2023 | RCT | South Korea | TKA | Intervention: 71.6 (6.8)  Control: 72.0 (6.1) | 172, 32 F | Preoperative education plus a module of realistic expectations delivered 1:1 | Duration not specified | Patient satisfaction | Pre-op: nil  Post-op: 12, 26 and >52 weeks |
| Nguyen et al., ^54^ | 2022 | RCT | France | TKA | 68.6 (8.0) | 262, 178 F | Group based multi-disciplinary prehabilitation and education. Involved 4 educational sessions covering different topics, 4 supervised exercise sessions, and a home-based exercise program | Program delivered at least 2 months before surgery, 2x weekly | Percentage of patients reaching functional independence at post-op day 4 and WOMAC | Pre-op: nil  Post-op: 26 and 52 weeks |
| Nunez et al., ^55^ | 2006 | RCT | Spain | TKA | Intervention: 72.59 (6.20)  Control: 69.45 (6.79) | 100, 71 F | Therapeutic education and functional readaptation + conventional (pharmacological) treatment. | 12-week program  Combination of individual and group sessions (groups 10 -12 patients) | WOMAC | Pre-op: 6 months post intervention  Post-op: unclear |
| Okpara et al., ^56^ | 2025 | Pilot | Canada | THA & TKA | 74 (7.5) | 69, 47 F | Exercise, protein and vitamin D supplements, and medication review. | 3-10 months  Exercise: 3x weekly  Supplements: daily  Medication review: once-off | Feasibility | Pre-op: 1 week  Post-op: 6 and 26 weeks |
| Oosting et al., ^57^ | 2012 | Pilot | Netherlands | THA | Intervention: 76.9 (6.3)  Control: 75.0 (6.3) | 30, 24 F | Exercise and functional: a preoperative, home-based program to train functional activities and walking capacity | 3–6-week program, 2x weekly (plus 4x weekly unsupervised) | Feasibility | Pre-op: 2-4 days  Post-op: discharge, 6 weeks |
| Parsons et al., ^58^ | 2013 | RCT | England | THA & TKA | 73 (7.21) | 336 (250 analysed), 154 F | Health maintenance clinic intervention (for symptom management) | Duration not specified | WOMAC | Pre-op: 2 weeks  Post-op: nil |
| Patane et al., ^59^ | 2025 | RCT | Italy | THA & TKA | Group 1: 67.7 (65.1, 70.2)  Group 2: 68.2 (65.6, 70.8) | 44, M:F ratio not specified | Tele-home-prehabilitation: electrostimulation vs home-based exercise | 4-week program, 3x weekly | 30 second sit-to-stand | Pre-op: unclear (end of the program)  Post-op: unclear (admission to and discharge from rehabilitation) |
| Percope de Andrade  et al., ^60^ | 2022 | RCT | Brazil | TKA | Intervention: 65.1 (9.3)  Control: 62.8 (11.8) | 79 (67 analysed), 53 F | MDT group education session with written and illustrated material | Once-off session | Range of motion at knee | Pre-op: nil  Post-op: 6 months |
| Przkora et al., ^61^ | 2021 | Pilot | United States | TKA | 67.2 (7.1) | 10, 7 F | Low-resistance exercise protocol with blood flow restriction (BFR) using a torniquet in the preoperative period | 4-week program, 2x weekly | Not specified | Pre-op: nil  Post-op: 2 weeks |
| Rahmatika et al., ^62^ | 2020 | Pilot | Indonesia | TKA | Intervention: 67 (57-75)  Control: 67.5 (56-70) | 16, 14 F | Theraband exercise program | 4-week program, 3x weekly | Not specified | Pre-op: 1 week  Post-op: 8 weeks |
| Risso et al., ^63^ | 2022 | RCT | Scotland | TKA | 71.1 (8.1) | 29 (21 analysed), 9 F | Acute prehabilitative neuromuscular exercise-conditioning | 1 week program, 3x weekly and 3x per day | Neuromuscular performance | Pre-op: 1 week  Post-op: nil |
| Rognsvag et al., ^64^ | 2024 | Feasibility | Norway | TKA | 63.8 (7.26) | 10 (did not include rehab post surgery group), 5 F | Osteoarthritis education, exercise therapy, internet-delivered CBT (10 modules) | 12-week program, 2x weekly exercise sessions | KOOS | Pre-op: 3, 6, and 12 months (patients doing prehab asked to delay surgery)  Post-op: nil |
| Rooks et al., ^65^ | 2006 | RCT | United States | THA & TKA | Intervention: THA: 65 (11) TKA: 65 (8)  Control: THA: 59 (7)  TKA: 69 (8) | 108, 60 F | Cardiovascular, strength, and flexibility training (water- and land-based) | 6-week program, 3x weekly | WOMAC | Pre-op: 1 week  Post-op: 8 and 26 weeks |
| Roxburgh et al., ^66^ | 2024 | RCT | New Zealand | THA & TKA | Heat: 66 (7)  HIIT: 71 (9)  Control: 67 (8) | 90 (78 analysed), 41 F | Heat therapy (hot-water immersion and light-resistance exercise sessions) or HIIT (exercise on either a cross-trainer or arm ergometer) | 12-week program, 3x weekly | Peak VO2 | Pre-op: unclear - “after 12 weeks of the intervention (or prior, if surgery was scheduled), both assessment sessions were repeated”  Post-op: nil |
| Sandell et al., ^67^ | 2008 | RCT | England | THA | Intervention: 70.33 (8.12)  Control: 65.8 (10.55) | 89, 41 F | Multidisciplinary assessment and intervention (physiotherapy, orthopaedic nurse specialist, nurse specialist in acute pain, and an occupational therapist) | Duration and frequency not specified | Arthritis Impact Measurement Score 2, Nottingham Health Profile | Pre-op: unclear - “pre surgery”  Post-op: nil |
| Savkin et al., ^68^ | 2021 | RCT | Turkey | TKA | Intervention: 64.1 (5.06)  Control: 64.25 (5.52) | 40, 3 F | Neuromuscular electrical stimulation | 6-week program, 5x daily | WOMAC | Pre-op: unclear - “6 weeks after baseline”  Post-op: 4 and 12 weeks |
| Saw et al., ^69^ | 2016 | RCT | South Africa | THA & TKA | 60.72 (5.54) | 74, 60 F | Physiotherapist-led exercise and education intervention | 6-week program, 1x weekly | Brief Pain Inventory | Pre-op: unclear –outcomes collected at 6 weeks, 12 weeks, and 6 months post-commencement of intervention  Post-op: unclear |
| Setiawati et al., ^70^ | 2020 | RCT | Indonesia | TKA | Intervention: 67 (range 57-75)  Control: 67.5 (range 56-70) | 16, 14 F | Theraband exercise program | 4-week program, 3x weekly | SF-36 | Pre-op: nil  Post-op: 8 weeks |
| Seward et al., ^71^ | 2025 | RCT | United States | THA & TKA | Intervention: 62.3 (10)  Control: 60.1 (8) | 60, 40 F | Telehealth dietician and mobile application | 12-week program, 1x weekly | Weight loss | Pre-op: unclear (reported 6 weeks and 12 weeks but unclear if relative to intervention or randomisation)  Post-op: unclear |
| Siggeirsdottir et al., ^72^ | 2005 | RCT | Iceland | THA | 68 (range 28–86) | 50, 26 F | Preoperative education and exercise + brochure | 4-week program | Not specified | Pre-op: 1 day  Post-op: 8, 16 and 26 weeks |
| Skoffer et al., ^73^ | 2016 | RCT | Denmark | TKA | Intervention: 70.7 (7.3)  Control: 70.1 (6.4) | 59, 36 F | Progressive resistance training in groups of 3 | 4-week program, 3x weekly | 30 second sit-to-stand | Pre-op: 1 week  Post-op: 1, 6 and 12 weeks |
| Skoffer et al., ^74^ | 2020 |  |  |  |  |  |  |  |  | Pre-op: 1 week  Post-op: 12 months |
| Soeters et al., ^75^ | 2018 | RCT | United States | THA & TKA | Intervention: 61 (9)  Control:  62 (8) | 126, 80 F | Preoperative physical therapy education + web-based microsite | 1x education session, 2 weeks access to microsite | Patient's readiness for discharge from physiotherapy | Pre-op: nil  Post-op: 4-6 weeks |
| Soni et al., ^76^ | 2012 | RCT | England | TKA | Intervention: 66.89 (9.82)  Control: 69.93 (7.85) | 56, 28 F | Supervised exercise (group based) plus acupuncture | Weekly sessions for 4 weeks, then fortnightly sessions for 4 weeks and monthly sessions until surgery | Oxford Knee Score | Pre-op: 6 and 12 weeks after intervention  Post-op: 12 weeks |
| Sun et al., ^77^ | 2023 | RCT | China | TKA | Intervention: 66.4 (8.3)  Control: 68.5 (7.9) | 100 (67 analysed), 46 F | High-intensity strength training combined with balance training | 4-week program, 5x weekly | Knee Society Score | Pre-op: unclear - “before surgery”  Post-op: 12 and 52 weeks |
| Svinoy et al., ^78^ | 2025 | RCT | Norway | THA | Intervention: 76.8±4.4  Control: 76.3±4.7 | 98, 64 F | Preoperative exercises and education program | 6- to 12-week program, 3-4x weekly | Gait speed on 40 meter fast-paced walk | Pre-op: 1 week post intervention  Post-op: 6, 12, 26, and 52 weeks |
| Swank et al., ^79^ | 2011 | RCT | United States | TKA | Intervention: 63.1 (7.3)  Control:  62.6 (7.6) | 71, 46 F | Exercise (resistance training using bands, flexibility, and step training) | 4–8-week program, 3x weekly | Not specified | Pre-op: 1 week  Post-op: nil |
| Terradas-Monllor et al., ^80^ | 2023 | Feasibility | Spain | TKA | Intervention 1: 72.52 (4.53)  Intervention 2: 71.14 (5.12)  Control: 72.67 (5.75) | 33, 24 F | Intervention 1:  3 home-based education sessions (pain neuroscience and coping skills)  Intervention 2: 8 home-based sessions of pre-operative multimodal physiotherapy | 3-week program | Pain Catastrophising Scale and feasibility | Pre-op: unclear - "post-treatment follow-up occurred 8 weeks after the baseline assessment point"  Post-op: nil |
| Terradas-Monllor et al., ^81^ | 2025 | RCT | Spain | TKA | Intervention 1: 66.7 (5.2)  Intervention 2: 60.6 (5.8)  Control: 59.4 (6.2) | 40, 29 F | Intervention 1:  3 home-based education sessions (pain neuroscience and coping skills)  Intervention 2: 8 home-based sessions of pre-operative | 3-week program | Pain Catastrophising Scale | Pre-op: unclear - "post-treatment follow-up occurred 8 weeks after the baseline assessment point"  Post-op: 4, 12, and 26 weeks |
| Tillu et al., ^82^ | 2001 | RCT | England | TKA | Intervention 1: 72 (range 53-90)  Intervention 2:  73 (range 64-92) | 44, 35 F | Intervention 1: unilateral acupuncture  Intervention 2: bilateral acupuncture | 6-week program, 1x weekly | Not specified | Pre-op: unclear – 2 and 6 months post intervention commencement  Post-op: nil |
| Tolk et al., ^83^ | 2021 | RCT | Netherlands | TKA | Intervention: 68.4 (8.7)  Control: 69.0 (10.1) | 204, 122 F | Preoperative education module on realistic expectations | Once-off session | Satisfaction (numerical rating scale) | Pre-op: nil  Post-op: 12 months |
| Topp et al., ^84^ | 2009 | RCT | United States | TKA | Intervention: 64.1 (7.05)  Control: 63.5 (6.68) | 54, 37 F | Mixed exercise (resistance, flexibility, step training) | 4-week program, 3x weekly | Not specified | Pre-op: 1 week  Post-op: 4 and 12 weeks |
| Tungtrongjit et al., ^85^ | 2012 | RCT | Thailand | TKA | Intervention: 63 (7.6)  Control: 65.9 (7.2) | 60, 50 F | Home program focused on quads strengthening | 3-week program, 3x daily | Knee pain (visual analogue scale) | Pre-op: nil  Post-op: 4, 12 and 26 weeks |
| Van Leeuwen et al., ^86^ | 2014 | Feasibility | Netherlands | TKA | Intervention: 71.8 (7.5)  Control: 69.5 (7.1) | 22, 10 F | Strength training | 6-week program, 2-3x weekly | Knee extension strength | Pre-op: 1 week  Post-op: 6 and 12 weeks |
| Villadsen et al.,  ^87^ | 2014 | RCT | Denmark | THA & TKA | 67 (8) | 165, 92 F | Neuromuscular supervised exercise programme - group setting (6-12 patients) | 8-week program, 2x weekly | HOOS/KOOS ADL | Pre-op: nil  Post-op: 6 and 12 weeks |
| Fernandes et al., ^88^ | 2017 |  |  |  |  |  |  |  |  | Pre-op: nil  Post-op: one year |
| Walls et al., ^89^ | 2010 | Pilot | Ireland | TKA | 65.4 (range 49 to 80) | 17, 11 F | Home-based unsupervised neuromuscular electrical stimulation for quads (isometric contraction) | 8-week program, first 2 weeks every second day, then 5x weekly for next 6 weeks | Not specified | Pre-op: unclear - “week 8 preoperatively”  Post-op: 6 and 12 weeks |
| Wang et al., ^90^ | 2002 | RCT | Australia | THA | Intervention: 68.3 (8.2)  Control: 65.7 (8.4) | 28, 18 F | Resistance exercise + hydrotherapy | 2x supervised clinic-based sessions and 2x home-based sessions per week for 8 weeks preop | 25 metre walk test | Pre-op: 1 week  Post-op: 3, 12 and 24 weeks |
| Williams et al., ^91^ | 2022 | RCT | Australia | THA & TKA | 66.4 (7.2) | 63, 34 F | Progressive exercise group classes (max of 5 participants) and group behaviour counselling sessions | 12-week program, 2x weekly | Daily physical activity | Pre-op: unclear - “6 months post baseline assessment”  Post-op: 6 months |
| Wilson et al., ^92^ | 2016 | RCT | Canada | TKA | Intervention: 67 (8) Control: 66 (8) | 143, 89 F | Education program: booklet, individual teaching session, phone support | Teaching session and booklet within 4 weeks of surgery, phone call week prior to surgery | Brief Pain Inventory Interference | Pre-op: nil  Post-op: day 1, 2 and 3 |
| Yaqub et al., ^93^ | 2024 | RCT | Pakistan | TKA | Intervention: 65.2±6.8  Control: 64.7±7.1 | 88, 56 F | Supervised exercise sessions | 4-week program, 3x weekly | Timed-Up-and-Go test | Pre-op: nil  Post-op: 6 and 12 weeks |
| Zdziechowski et al., ^94^ | 2024 | RCT | Poland | THA | Centre-based: 64.1 (10.5)  Home-based: 69.0 (8.4)  Control: 68.3 (9.4) | 61 (50 analysed), 33 F | Compared centre-based program with home-based training (isometric and stretching exercises) | 3-week program, 5x weekly | Not specified | Pre-op: nil  Post-op: approximately 2 and 4 weeks |

Footnote: *Where primary outcome was not specified there was evidence of a formal sample size calculation performed for an outcome measure

**Pre-op timepoint refers to post prehabilitation intervention and before surgery

*Abbreviations: ASA: American Society of Anesthesiologists; BMI: body mass index; CBT: cognitive behavioural therapy; CDI: Caton Deschamps Index; EQ-5D-3L: European Quality of Life- 5 Dimension 3 Level version; F: female; HADS: Hospital Anxiety and Depression Scale; HIIT: high intensity interval training; HOOS: Hip disabilities and Osteoarthritis Outcome Score; KOOS-ADL: Knee injury and Osteoarthritis Outcome Score – Activities of Daily Living; MADRS: Montgomery and Aasberg Depression Rating Scale; MAT-sf: Mobility Assessment Tool-short form; MDT: multi-disciplinary team; NSQIP: National Surgical Quality Improvement Project; OHS: Oxford Hip Score; PCS: Pain Catastrophising Scale; post-op: post-operatively; pre-op: pre-operatively; PROMIS: Patient Reported Outcomes Measurement Information System; RAPT: Risk Assessment and Prediction Tool; RCT: randomised controlled trial; SF-36: 36-item short form survey; THA: total hip arthroplasty; TKA: total knee arthroplasty; VAS: visual analogue scale; VO2: maximum oxygen consumption; WOMAC: Western Ontario and McMaster Universities Arthritis Index.*

**Patient-Reported Outcomes**

**Concepts of interest**

Disease-specific assessments

Functional capacity

Physical activity

Quality of life

Disease-specific QoL

Fear avoidance/catastrophising

Pain

Anxiety and depression

Satisfaction

Beliefs

Self-rated health

Education/behaviour change

**eFigure 1.** Patient-Reported Outcome Measurement Tools. The number of times each tool was used as an outcome measure across trials is denoted within each section. The colours represent different concepts of interest within the Patient-Reported outcome domain.

*Abbreviations: ADL: Activities of Daily Living; APAIS: Amsterdam Preoperative Anxiety and Information Scale; AQoL: Assessment of Quality of Life; BMQ: Beliefs about Medicines Questionnaire; CES-D: Centre for Epidemiologic Studies Depression Scale; DVPRS: Defence and Veterans Pain Rating Scale; EQ-5D: European Quality of Life- 5 Dimension; FABQ: Fear Avoidance Belief Questionnaire; GAD: Generalised Anxiety Disorder; GDS: Geriatric Depression Scale; HADS: Hospital Anxiety and Depression Scale; heiQ: health education impact questionnaire; HOOS: Hip disability and Osteoarthritis Outcome Score; HPSI: Hospital Patient Satisfaction Inventory; IAP: Impairment, Activity, Participation; ICOAP: Intermittent and Constant OA Pain scale; ICECAP-O: ICEpop CAPability measure for Older people; IPAQ: International Physical Activity Questionnaire; JKOM: Japanese Knee Outcome Measure; KOOS: Knee disability and Osteoarthritis Outcome Score; LAPAQ: Longitudinal Ageing Study Amsterdam Physical Activity Questionnaire; LEFS: Lower Extremity Functional Scale; MAT: Mobility Assessment Tool; MODEMS: Musculoskeletal Outcomes Data Evaluation and Management System; MOST: Measure of Older Adults Sedentary Time; NHANES: National Health and Nutrition Examination Survey; NRS: Numerical Rating Scale; OEE: Outcome Expectations for Exercise; OHS: Oxford hip score; OKS: Oxford Knee Score; PGIC: Patient Global Impression of Change; PSFS: Patient Specific Functional Scale; PROMIS: Patient Reported Outcomes Measurement Information System; QoL: Quality of Life; SF-36/SF-12: 36-item/12-item short form survey; SRBAI: Self-Report Behavioural Automaticity Index; VAS: Visual Analogue Scale; WHO: World Health Organisation; WOMAC: Western Ontario and McMaster Universities Arthritis Index.*

**Concepts of interest**

Walking tests

Muscle strength

Range of motion

Cardiorespiratory fitness

Physical activity

Balance

Functional tests

**Performance-Based Outcomes**

**eFigure 2.** Performance-Based Outcome Measurement Tools. The number of times each tool was used as an outcome measure across trials is denoted within each section. The colours represent different concepts of interest within the Performance-Based outcome domain.

*Abbreviations: 2MWT: 2-Minute Walk Test; 4MWT: 4-Minute Walk Test; 6MWT: 6-Minute Walk Test; SPPB: Short Physical Performance Battery; STS: Sit To Stand; VO2: maximum oxygen consumption*

**Observer-Reported Outcomes**

**Concepts of interest**

Anthropometrics

Body composition

Vital signs

Healthcare utilisation

Medication usage

Mortality

**eFigure 3.** Observer-Reported Outcome Measurement Tools. The number of times each tool was used as an outcome measure across trials is denoted within each section. The colours represent different concepts of interest within the Observer-Reported outcome domain.

*Abbreviations: BMI: Body Mass Index; ED: Emergency Department; ICU: Intensive Care Unit; NSAIDs: Non-Steroidal Anti-Inflammatory Drugs; NSQIP: National Surgical Quality Improvement Program*

**Concepts of interest**

Post-operative complications

Disease-specific assessments

Functional independence

Cognitive function

Discharge readiness

Frailty

**Clinician-Reported Outcomes**

**eFigure 4.** Clinician-Reported Outcome Measurement Tools. The number of times each tool was used as an outcome measure across trials is denoted within each section. The colours represent different concepts of interest within the Clinician-Reported outcome domain.

*Abbreviations: ADLs: Activities of Daily Living; KSS: Knee Society Score; MoCA: Montreal Cognitive Assessment; NSQIP: National Surgical Quality Improvement Program*

**Concepts of interest**

Glucose markers

Inflammatory markers

Plasma proteins

Lipid markers

Other blood products

**eFigure 5.** Biomarker Outcome Measurement Tools. The number of times each tool was used as an outcome measure across trials is denoted within each section. The colours represent different concepts of interest within the Biomarker outcome domain.

*CRP: C-reactive protein; FBG: Fasting Blood Glucose; HbA1c: glycated haemoglobin; HOMA: Homeostatic Model Assessment; IL-6: interleukin-6; TNFα: tumor necrosis factor alpha*

**References**

1. Amprachim SE, Vlamis J, Vlami MJ, et al. The Effect of Preoperative Information and Education on the Clinical Outcome of Total Hip Arthroplasty: A Prospective, Randomized Trial. *Cureus* 2024; 16: e73841. DOI: 10.7759/cureus.73841.

2. Aunger JA, Greaves CJ, Davis ET, et al. A novel behavioural INTErvention to REduce Sitting Time in older adults undergoing orthopaedic surgery (INTEREST): results of a randomised-controlled feasibility study. *Aging Clinical and Experimental Research* 2020; 32: 2565-2585. DOI: 10.1007/s40520-020-01475-6.

3. Beaupre LA, Lier D, Davies DM and Johnston DB. The effect of a preoperative exercise and education program on functional recovery, health related quality of life, and health service utilization following primary total knee arthroplasty. *Journal of rheumatology* 2004; 31: 1166‐1173. Journal article.

4. Berge DJ, Dolin SJ, Williams AC and Harman R. Pre-operative and post-operative effect of a pain management programme prior to total hip replacement: A randomized controlled trial. *Pain* 2004; 110: 33-39. DOI: 10.1016/j.pain.2004.03.002.

5. Bergin C, Speroni KG, Travis T, et al. Effect of preoperative incentive spirometry patient education on patient outcomes in the knee and hip joint replacement population. *Journal of Perianesthesia Nursing* 2014; 29: 20-27. DOI: 10.1016/j.jopan.2013.01.009.

6. Bertram W, Penfold C, Glynn J, et al. REST: a preoperative tailored sleep intervention for patients undergoing total knee replacement - feasibility study for a randomised controlled trial. *BMJ Open* 2024; 14: e078785. 20240320. DOI: 10.1136/bmjopen-2023-078785.

7. Birch S, Stilling M, Mechlenburg I and Hansen TB. No effect of cognitive behavioral patient education for patients with pain catastrophizing before total knee arthroplasty: a randomized controlled trial. *Acta Orthopaedica* 2020; 91: 98-103. DOI: 10.1080/17453674.2019.1694312.

8. Blasco J-M, Acosta-Ballester Y, Martinez-Garrido I, et al. The effects of preoperative balance training on balance and functional outcome after total knee replacement: a randomized controlled trial. *Clinical Rehabilitation* 2020; 34: 182-193. DOI: 10.1177/0269215519880936.

9. Brown K, Loprinzi PD, Brosky JA and Topp R. Prehabilitation influences exercise-related psychological constructs such as self-efficacy and outcome expectations to exercise. *Journal of Strength and Conditioning Research* 2014; 28: 201-209. DOI: 10.1519/JSC.0b013e318295614a.

10. Brown K, Top R, Brosky JA and Scott Lajoie A. Prehabilitation and quality of life three months after total knee arthroplasty: A pilot study. *Perceptual and Motor Skills* 2012; 115: 765-774. DOI: 10.2466/15.06.10.PMS.115.6.765-774.

11. Buvanendran A, Sremac AC, Merriman PA, et al. Preoperative cognitive-behavioral therapy for reducing pain catastrophizing and improving pain outcomes after total knee replacement: a randomized clinical trial. *Regional Anesthesia and Pain Medicine* 2021; 46: 313-321. DOI: 10.1136/rapm-2020-102258.

12. Calatayud J, Casaña J, Ezzatvar Y, et al. High-intensity preoperative training improves physical and functional recovery in the early post-operative periods after total knee arthroplasty: a randomized controlled trial. *Knee Surgery, Sports Traumatology, Arthroscopy* 2017; 25: 2864-2872. DOI: 10.1007/s00167-016-3985-5.

13. Casana J, Calatayud J, Ezzatvar Y, et al. Preoperative high-intensity strength training improves postural control after TKA: randomized-controlled trial. *Knee Surgery, Sports Traumatology, Arthroscopy* 2019; 27: 1057-1066. DOI: 10.1007/s00167-018-5246-2.

14. Cannata F, Laudisio A, Russo F, et al. Weight Loss in Patients Waiting for Total Hip Arthroplasty: Fiber-Enriched High Carbohydrate Diet Improves Hip Function and Decreases Pain before Surgery. *Journal of Clinical Medicine* 2021; 10. DOI: 10.3390/jcm10184203.

15. Cavill S, McKenzie K, Munro A, et al. The effect of prehabilitation on the range of motion and functional outcomes in patients following the total knee or hip arthroplasty: A pilot randomized trial. *Physiotherapy Theory and Practice* 2016; 32: 262-270. DOI: 10.3109/09593985.2016.1138174.

16. Chen G, Yu D, Wang Y, et al. A Prospective Randomized Controlled Trial Assessing the Impact of Preoperative Combined with Postoperative Progressive Resistance Training on Muscle Strength, Gait, Balance and Function in Patients Undergoing Total Hip Arthroplasty. *Clin Interv Aging* 2024; 19: 745-760. 20240508. DOI: 10.2147/cia.S453117.

17. Crotty M, Prendergast J, Battersby MW, et al. Self-management and peer support among people with arthritis on a hospital joint replacement waiting list: a randomised controlled trial. *Osteoarthritis Cartilage* 2009; 17: 1428-1433. 20090521. DOI: 10.1016/j.joca.2009.05.010.

18. das Nair R, Mhizha-Murira JR, Anderson P, et al. Home-based pre-surgical psychological intervention for knee osteoarthritis (HAPPiKNEES): a feasibility randomized controlled trial. *Clinical Rehabilitation* 2018; 32: 777‐789. Journal article. DOI: 10.1177/0269215518755426.

19. De Luis D, Izaola O, García Alonso M, et al. Effect of a commercial hypocaloric diet in weight loss and post surgical morbidities in obese patients with chronic arthropathy, a randomized clinical trial. *European Review for Medical & Pharmacological Sciences* 2012; 16.

20. Doiron-Cadrin P, Kairy D, Vendittoli PA, et al. Feasibility and preliminary effects of a tele-prehabilitation program and an in-person prehablitation program compared to usual care for total hip or knee arthroplasty candidates: a pilot randomized controlled trial. *Disability and Rehabilitation* 2020; 42: 989‐998. Journal article. DOI: 10.1080/09638288.2018.1515992.

21. Dominguez-Navarro F, Silvestre-Munoz A, Igual-Camacho C, et al. A randomized controlled trial assessing the effects of preoperative strengthening plus balance training on balance and functional outcome up to 1 year following total knee replacement. *Knee Surgery, Sports Traumatology, Arthroscopy*  2021; 29: 838-848. DOI: 10.1007/s00167-020-06029-x.

22. Dowsey M, Castle D, Knowles S, et al. The effect of mindfulness training prior to total joint arthroplasty on post-operative pain and physical function: A randomised controlled trial. *Complementary Therapies in Medicine* 2019; 46: 195-201. DOI: 10.1016/j.ctim.2019.08.010.

23. Evgeniadis G, Beneka A, Malliou P, et al. Effects of pre- or postoperative therapeutic exercise on the quality of life, before and after total knee arthroplasty for osteoarthritis. *Journal of Back and Musculoskeletal Rehabilitation* 2008; 21: 161-169. DOI: 10.3233/BMR-2008-21303.

24. Ferrara PE, Rabini A, Aprile I, et al. Effect of pre-operative physiotherapy in patients with end-stage osteoarthritis undergoing hip arthroplasty. *Clinical Rehabilitation* 2008; 22: 977-986. DOI: 10.1177/0269215508094714.

25. Franz A, Ji S, Bittersohl B, et al. Impact of a Six-Week Prehabilitation With Blood-Flow Restriction Training on Pre- and Postoperative Skeletal Muscle Mass and Strength in Patients Receiving Primary Total Knee Arthroplasty. *Frontiers in Physiology* 2022; 13: 881484. DOI: 10.3389/fphys.2022.881484.

26. Gilbey HJ, Ackland TR, Tapper J and Wang AW. Perioperative exercise improves function following total hip arthroplasty: A randomized controlled trial. *Journal of Musculoskeletal Research* 2003; 7: 111-123. DOI: 10.1142/S0218957703001046.

27. Giraudet-Le Quintrec J, Coste J, Vastel L, et al. Positive effect of patient education for hip surgery: a randomized trial. *Clinical Orthopaedics & Related Research®* 2003; 414: 112-120.

28. Granicher P, Mulder L, Lenssen T, et al. Exercise- and education-based prehabilitation before total knee arthroplasty: a pilot study. *Journal of Rehabilitation Medicine* 2024; 56: jrm18326. DOI: 10.2340/jrm.v56.18326.

29. Granicher P, Stoggl T, Fucentese SF, et al. Preoperative exercise in patients undergoing total knee arthroplasty: a pilot randomized controlled trial. *Archives of Physiotherapy* 2020; 10: 13. DOI: 10.1186/s40945-020-00085-9.

30. Gstoettner M, Raschner C, Dirnberger E, et al. Preoperative proprioceptive training in patients with total knee arthroplasty. *KNEE* 2011; 18: 265-270. DOI: 10.1016/j.knee.2010.05.012.

31. Haslam R. A comparison of acupuncture with advice and exercises on the symptomatic treatment of osteoarthritis of the hip–a randomised controlled trial. *Acupuncture in Medicine* 2001; 19: 19-26.

32. Hermann A, Holsgaard-Larsen A, Zerahn B, et al. Preoperative progressive explosive-type resistance training is feasible and effective in patients with hip osteoarthritis scheduled for total hip arthroplasty--a randomized controlled trial. *Osteoarthritis and Cartilage* 2016; 24: 91-98. DOI: 10.1016/j.joca.2015.07.030.

33. Hoogeboom TJ, Dronkers JJ, van den Ende CH, et al. Preoperative therapeutic exercise in frail elderly scheduled for total hip replacement: a randomized pilot trial. *Clinical Rehabilitation* 2010; 24: 901-910.

34. Huang SW, Chen PH and Chou YH. Effects of a preoperative simplified home rehabilitation education program on length of stay of total knee arthroplasty patients. *Orthopaedics & Traumatology, Surgery & Research : OTSR* 2012; 98: 259-264. DOI: 10.1016/j.otsr.2011.12.004.

35. Huber EO, Roos EM, Meichtry A, et al. Effect of preoperative neuromuscular training (NEMEX-TJR) on functional outcome after total knee replacement: an assessor-blinded randomized controlled trial. *BMC Musculoskeletal Disorders* 2015; 16: 101. DOI: 10.1186/s12891-015-0556-8.

36. Husted RS, Troelsen A, Husted H, et al. Knee-extensor strength, symptoms, and need for surgery after two, four, or six exercise sessions/week using a home-based one-exercise program: a randomized dose-response trial of knee-extensor resistance exercise in patients eligible for knee replacement (the QUADX-1 trial). *Osteoarthritis Cartilage* 2022; 30: 973-986. 20220409. DOI: 10.1016/j.joca.2022.04.001.

37. Ibrahim MI, Hussein AZ and Donatelli R. Preoperative rehabilitation does not affect quality of life and functional outcomes in patients following total hip or knee arthroplasty. *Bull Fac Ph Th Cairo Univ* 2014; 19.

38. Jahic D, Omerovic D, Tanovic AT, et al. The Effect of Prehabilitation on Postoperative Outcome in Patients Following Primary Total Knee Arthroplasty. *Medical Archives (Sarajevo, Bosnia and Herzegovina)* 2018; 72: 439-443. DOI: 10.5455/medarh.2018.72.439-443.

39. Jepson P, Sands G, Beswick AD, et al. A feasibility randomised controlled trial of pre-operative occupational therapy to optimise recovery for patients undergoing primary total hip replacement for osteoarthritis (PROOF-THR). *Clinical Rehabilitation* 2016; 30: 156-166. DOI: 10.1177/0269215515576811.

40. Jintana R, Suparb A-u, Pomtip M, et al. The Effectiveness of Preoperative Quadriceps Exercise and Diet Control Program for Older Adults Waiting for Total Knee Arthroplasty: a Randomized Controlled Trial. *Pacific Rim International Journal of Nursing Research* 2020; 24: 485‐501. Journal article.

41. Jorgensen S, Aagaard P, Bohn M, et al. The Effect of Blood Flow Restriction Exercise Prior to Total Knee Arthroplasty on Postoperative Physical Function, Lower Limb Strength and Patient-Reported Outcomes: A Randomized Controlled Trial (vol 34, e14750, 2024). *Scandinavian Journal of Medicine and Science in Sports* 2024; 34. DOI: 10.1111/sms.14776.

42. Kaynar AM, Zharichenko N, Wasan AD and Chelly JE. Telemedicine-based digital cognitive behavioral intervention for perioperative anxiety and depression for total knee arthroplasty. *Journal of Pain & Relief* 2023; 12.

43. Khalid T, Ben-Shlomo Y, Bertram W, et al. Prehabilitation for frail patients undergoing hip and knee replacement in the UK: joint PREP feasibility study for a randomised controlled trial. *BMJ Open* 2024; 14: e084678. Journal article. DOI: 10.1136/bmjopen-2024-084678.

44. Kim S, Hsu FC, Groban L, et al. A pilot study of aquatic prehabilitation in adults with knee osteoarthritis undergoing total knee arthroplasty - short term outcome. *BMC Musculoskeletal Disorders* 2021; 22: 388. Journal article. DOI: 10.1186/s12891-021-04253-1.

45. Kubo Y, Fujita D, Sugiyama S, et al. Safety and Effects of a Four-Week Preoperative Low-Load Resistance Training With Blood Flow Restriction on Pre- and Postoperative Quadriceps Strength in Patients Undergoing Total Knee Arthroplasty: A Single-Blind Randomized Controlled Trial. *Cureus* 2024; 16: e64466. DOI: 10.7759/cureus.64466.

46. Leal‐Blanquet J, Alentorn‐Geli E, Ginés‐Cespedosa A, et al. Effects of an educational audiovisual videodisc on patients’ pre‐operative expectations with total knee arthroplasty: a prospective randomized comparative study. *Knee Surgery, Sports Traumatology, Arthroscopy* 2013; 21: 2595-2602.

47. Liljensoe A, Laursen JO, Bliddal H, et al. Weight Loss Intervention Before Total Knee Replacement: A 12-Month Randomized Controlled Trial. *Scandinavian journal of surgery : SJS : official organ for the Finnish Surgical Society and the Scandinavian Surgical Society* 2021; 110: 3-12. DOI: 10.1177/1457496919883812.

48. Lluch E, Duenas L, Falla D, et al. Preoperative Pain Neuroscience Education Combined With Knee Joint Mobilization for Knee Osteoarthritis: A Randomized Controlled Trial. *The Clinical Journal of Pain* 2018; 34: 44-52. DOI: 10.1097/AJP.0000000000000511.

49. Matassi F, Duerinckx J, Vandenneucker H and Bellemans J. Range of motion after total knee arthroplasty: the effect of a preoperative home exercise program. *Knee Surgery, Sports Traumatology, Arthroscopy* 2014; 22: 703-709. DOI: 10.1007/s00167-012-2349-z.

50. McGregor AH, Rylands H, Owen A, et al. Does preoperative hip rehabilitation advice improve recovery and patient satisfaction? *Journal of Arthroplasty* 2004; 19: 464‐468. Journal article. DOI: 10.1016/j.arth.2003.12.074.

51. McKay C, Prapavessis H and Doherty T. The effect of a prehabilitation exercise program on quadriceps strength for patients undergoing total knee arthroplasty: a randomized controlled pilot study. *PM & R : the journal of Injury, Function, and Rehabilitation* 2012; 4: 647-656. DOI: 10.1016/j.pmrj.2012.04.012.

52. Medina-Garzón M. Effectiveness of a nursing intervention to diminish preoperative anxiety in patients programmed for knee replacement surgery: preventive controlled and randomized clinical trial. *Investigacion y Educacion en Enfermeria* 2019; 37.

53. Nam HS, Yoo HJ, Ho JPY, et al. Preoperative education on realistic expectations improves the satisfaction of patients with central sensitization after total knee arthroplasty: a randomized-controlled trial. *Knee Surgery, Sports Traumatology, Arthroscopy* 2023. DOI: 10.1007/s00167-023-07487-9.

54. Nguyen C, Boutron I, Roren A, et al. Effect of Prehabilitation Before Total Knee Replacement for Knee Osteoarthritis on Functional Outcomes: a Randomized Clinical Trial. *JAMA Network Open* 2022; 5: e221462. Journal article. DOI: 10.1001/jamanetworkopen.2022.1462.

55. Nuñez M, Nuñez E, Segur JM, et al. The effect of an educational program to improve health-related quality of life in patients with osteoarthritis on waiting list for total knee replacement: a randomized study. *Osteoarthritis Cartilage* 2006; 14: 279-285. 20051123. DOI: 10.1016/j.joca.2005.10.002.

56. Okpara C, Negm A, Adachi JD, et al. Getting fit for hip and knee replacement: The Fit-Joints multimodal intervention for frail patients with osteoarthritis - a pilot randomized controlled trial. *The Journal of Frailty & Aging* 2025; 14: 100028. DOI: 10.1016/j.tjfa.2025.100028.

57. Oosting E, Jans MP, Dronkers JJ, et al. Preoperative home-based physical therapy versus usual care to improve functional health of frail older adults scheduled for elective total hip arthroplasty: a pilot randomized controlled trial. *Archives of Physical Medicine and Rehabilitation* 2012; 93: 610-616. DOI: 10.1016/j.apmr.2011.11.006.

58. Parsons G, Jester R and Godfrey H. A randomised controlled trial to evaluate the efficacy of a health maintenance clinic intervention for patients undergoing elective primary total hip and knee replacement surgery. *International Journal of Orthopaedic & Trauma Nursing* 2013; 17: 171‐179. Journal article. DOI: 10.1016/j.ijotn.2013.07.004.

59. Patane P, Carnevale Pellino V, Febbi M, et al. Effects of a Tele-Prehabilitation Program with Indirect Electrostimulation Compared to Home-Based Exercise in Patients Eligible for Lower Limb Arthroplasty: A Randomized Controlled Trial. *Journal of Clinical Medicine* 2025; 14. DOI: 10.3390/jcm14041356.

60. Percope de Andrade MA, Moreira de Abreu Silva G, de Oliveira Campos TV, et al. A new methodology for patient education in total knee arthroplasty: a randomized controlled trial. *European Journal of Orthopaedic Surgery & Traumatology* 2022; 32: 107-112. DOI: 10.1007/s00590-021-02936-y.

61. Przkora R, Sibille K, Victor S, et al. Blood flow restriction exercise to attenuate postoperative loss of function after total knee replacement: A randomized pilot study. *European Journal of Translational Myology* 2021; 31: 9932. DOI: 10.4081/ejtm.2021.9932.

62. Rahmatika R, Novriansyah R and Indriastuti L. The effects of prehabilitation exercise using resistance bands on functional performance in total knee replacement. *The Hip and Knee Journal* 2020; 1: 7-16.

63. Risso AM, van der Linden ML, Bailey A, et al. Exploratory insights into novel prehabilitative neuromuscular exercise-conditioning in total knee arthroplasty. *BMC Musculoskeletal Disorders* 2022; 23: 547. DOI: 10.1186/s12891-022-05444-0.

64. Rognsvåg T, Bergvad IB, Furnes O, et al. Exercise therapy, education, and cognitive behavioral therapy alone, or in combination with total knee arthroplasty, in patients with knee osteoarthritis: a randomized feasibility study. *Pilot and Feasibility Studies* 2024; 10. DOI: 10.1186/s40814-024-01470-y.

65. Rooks DS, Huang J, Bierbaum BE, et al. Effect of preoperative exercise on measures of functional status in men and women undergoing total hip and knee arthroplasty. *Arthritis and Rheumatism* 2006; 55: 700-708. DOI: 10.1002/art.22223.

66. Roxburgh B, Campbell HA, Cotter J, et al. Maintenance of Preoperative Fitness by Home-Based Prehabilitation Following Supervised Prehabilitation in Patients Awaiting Total Hip or Knee Arthroplasty. *International Journal of Research in Exercise Physiology* 2024; 19: 57-77.

67. Sandell C. A multidisciplinary assessment and intervention for patients awaiting total hip replacement to improve their quality of life. *Journal of Orthopaedic Nursing* 2008; 12: 26-34. DOI: 10.1016/j.joon.2007.11.002.

68. Savkin R, Buker N and Gungor HR. The effects of preoperative neuromuscular electrical stimulation on the postoperative quadriceps muscle strength and functional status in patients with fast-track total knee arthroplasty. *Acta Orthopaedica Belgica* 2021; 87: 735-744. DOI: 10.52628/87.4.19.

69. Saw MM, Kruger-Jakins T, Edries N and Parker R. Significant improvements in pain after a six-week physiotherapist-led exercise and education intervention, in patients with osteoarthritis awaiting arthroplasty, in South Africa: a randomised controlled trial. *BMC Musculoskeletal Disorders* 2016; 17: 236. DOI: 10.1186/s12891-016-1088-6.

70. Setiawati E, Kusumadewi J and Tjandra R. Effect of Prehabilitation Exercise Using Resistance Bands on Quality of Life in Patients Undergoing Total Knee Replacement. *Indonesian Journal of Physical Medicine and Rehabilitation* 2020; 9: 29-39.

71. Seward MW, Liimakka AP, Jamison MP, et al. Weight Loss Before Total Joint Arthroplasty Using a Remote Dietitian and a Mobile Application: A Multicenter Randomized Controlled Trial. *The Journal of Bone and Joint Surgery American volume* 2025; 107: 910-918. DOI: 10.2106/JBJS.24.00838.

72. Siggeirsdottir K, Olafsson O, Jonsson H, et al. Short hospital stay augmented with education and home-based rehabilitation improves function and quality of life after hip replacement: randomized study of 50 patients with 6 months of follow-up. *Acta Orthopaedica* 2005; 76: 555-562. DOI: 10.1080/17453670510041565.

73. Skoffer B, Maribo T, Mechlenburg I, et al. Efficacy of Preoperative Progressive Resistance Training on Postoperative Outcomes in Patients Undergoing Total Knee Arthroplasty. *Arthritis Care & Research* 2016; 68: 1239-1251. DOI: 10.1002/acr.22825.

74. Skoffer B, Maribo T, Mechlenburg I, et al. Efficacy of preoperative progressive resistance training in patients undergoing total knee arthroplasty: 12-month follow-up data from a randomized controlled trial. *Clinical Rehabilitation* 2020; 34: 82-90. DOI: 10.1177/0269215519883420.

75. Soeters R, White PB, Murray-Weir M, et al. Preoperative Physical Therapy Education Reduces Time to Meet Functional Milestones After Total Joint Arthroplasty. *Clinical Orthopaedics and Related Research* 2018; 476: 40-48. DOI: 10.1007/s11999.0000000000000010.

76. Soni A, Joshi A, Mudge N, et al. Supervised exercise plus acupuncture for moderate to severe knee osteoarthritis: a small randomised controlled trial. *Acupuncture in Medicine : Journal of the British Medical Acupuncture Society* 2012; 30: 176-181. DOI: 10.1136/acupmed-2012-010128.

77. Sun J, Shan Y, Wu L, et al. Preoperative high-intensity strength training combined with balance training can improve early outcomes after total knee arthroplasty. *Journal ofOorthopaedic Surgery andRresearch* 2023; 18: 1‐9. Journal article. DOI: 10.1186/s13018-023-04197-3.

78. Svinoy O-E, Nordbo JV, Pripp AH, et al. The effect of prehabilitation for older patients awaiting total hip replacement. A randomized controlled trial with long-term follow up. *BMC Musculoskeletal Disorders* 2025; 26: 227. DOI: 10.1186/s12891-025-08468-4.

79. Swank AM, Joseph BK, Wendy B, et al. Prehabilitation before total knee arthroplasty increases strength and function in older adults with severe osteoarthritis. *Journal of Strength and Conditioning Research* 2011; 25: 318-325. DOI: 10.1519/JSC.0b013e318202e431.

80. Terradas-Monllor M, Ochandorena-Acha M, Beltran-Alacreu H, et al. A feasibility study of home-based preoperative multimodal physiotherapy for patients scheduled for a total knee arthroplasty who catastrophize about their pain. *Physiotherapy Theory and Practice* 2023; 39: 1606-1625. DOI: 10.1080/09593985.2022.2044423.

81. Terradas-Monllor M, Beltran-Alacreu H, Ochandorena-Acha M, et al. Preoperative Home-Based Multimodal Physiotherapy in Patients Scheduled for a Knee Arthroplasty Who Catastrophize About Their Pain: A Randomized Controlled Trial. *Journal of Clinical Medicine* 2025; 14. DOI: 10.3390/jcm14010268.

82. Tillu A, Roberts C and Tillu S. Unilateral versus bilateral acupuncture on knee function in advanced osteoarthritis of the knee–a prospective randomised trial. *Acupuncture in Medicine* 2001; 19: 15-18.

83. Tolk JJ, Janssen RPA, Haanstra TM, et al. The influence of expectation modification in knee arthroplasty on satisfaction of patients: a randomized controlled trial. *The Bone & Joint Journal* 2021; 103-B: 619-626. DOI: 10.1302/0301-620X.103B4.BJJ-2020-0629.R3.

84. Topp R, Swank AM, Quesada PM, et al. The effect of prehabilitation exercise on strength and functioning after total knee arthroplasty. *PM & R : The Journal of Injury, Function, and Rehabilitation* 2009; 1: 729-735. DOI: 10.1016/j.pmrj.2009.06.003.

85. Tungtrongjit Y, Weingkum P and Saunkool P. The effect of preoperative quadriceps exercise on functional outcome after total knee arthroplasty. *Journal of the Medical Association of Thailand = Chotmaihet thangphaet* 2012; 95 Suppl 10: S58-66.

86. van Leeuwen DM, de Ruiter CJ, Nolte PA and de Haan A. Preoperative strength training for elderly patients awaiting total knee arthroplasty. *Rehabilitation Research and Practice* 2014; 2014: 462750. DOI: 10.1155/2014/462750.

87. Villadsen A, Overgaard S, Holsgaard-Larsen A, et al. Postoperative effects of neuromuscular exercise prior to hip or knee arthroplasty: a randomised controlled trial. *Annals of the Rheumatic Diseases* 2014; 73: 1130-1137. DOI: 10.1136/annrheumdis-2012-203135.

88. Fernandes L, Roos EM, Overgaard S, et al. Supervised neuromuscular exercise prior to hip and knee replacement: 12-month clinical effect and cost-utility analysis alongside a randomised controlled trial. *BMC Musculoskeletal Disorders* 2017; 18: 5. DOI: 10.1186/s12891-016-1369-0.

89. Walls RJ, McHugh G, O'Gorman DJ, et al. Effects of preoperative neuromuscular electrical stimulation on quadriceps strength and functional recovery in total knee arthroplasty. A pilot study. *BMC Musculoskeletal Disorders* 2010; 11: 119. DOI: 10.1186/1471-2474-11-119.

90. Wang AW, Gilbey HJ and Ackland TR. Perioperative exercise programs improve early return of ambulatory function after total hip arthroplasty: a randomized, controlled trial. *American journal of physical medicine & rehabilitation* 2002; 81: 801-806.

91. Williams AD, O'Brien J, Mulford J, et al. Effect of combined exercise training and behaviour change counselling versus usual care on physical activity in patients awaiting hip and knee arthroplasty: A randomised controlled trial. *Osteoarthritis and Cartilage Open* 2022; 4: 100308. DOI: 10.1016/j.ocarto.2022.100308.

92. Wilson RA, Watt-Watson J, Hodnett E and Tranmer J. A Randomized Controlled Trial of an Individualized Preoperative Education Intervention for Symptom Management After Total Knee Arthroplasty. *Orthopedic Nursing* 2016; 35: 20-29. DOI: 10.1097/NOR.0000000000000210.

93. Yaqub A, Ahmed I, Zia M, et al. Impact of preoperative rehabilitation on functional outcomes following total knee arthroplasty: A randomized controlled trial. *Journal of Population Therapeutics and Clinical Pharmacology* 2024; 31: 1066. DOI: 10.53555/jptcp.v31i8.7532.

94. Zdziechowski A, Zdziechowska M, Rysz J and Woldanska-Okonska M. The Effectiveness of Preoperative Outpatient and Home Rehabilitation and the Impact on the Results of Hip Arthroplasty: Introductory Report. *Healthcare (Basel, Switzerland)* 2024; 12. DOI: 10.3390/healthcare12030327.
